# Nasal Immunization With Small Molecule Mast Cell Activators Enhance Immunity to Co-Administered Subunit Immunogens

**DOI:** 10.3389/fimmu.2021.730346

**Published:** 2021-09-10

**Authors:** Brandi T. Johnson-Weaver, Hae Woong Choi, Hang Yang, Josh A. Granek, Cliburn Chan, Soman N. Abraham, Herman F. Staats

**Affiliations:** ^1^Pathology Department, School of Medicine, Duke University, Durham, NC, United States; ^2^Biostatistics and Bioinformatics Department, School of Medicine, Duke University, Durham, NC, United States; ^3^Department of Immunology, School of Medicine, Duke University, Durham, NC, United States; ^4^Department of Molecular Genetics and Microbiology, Duke University, Durham, NC, United States; ^5^Duke Human Vaccine Institute, Duke University, Durham, NC, United States

**Keywords:** vaccine adjuvants, nasal vaccine, mast cells, small molecule adjuvants, adjuvant discovery

## Abstract

Mast cell activators are a novel class of mucosal vaccine adjuvants. The polymeric compound, Compound 48/80 (C48/80), and cationic peptide, Mastoparan 7 (M7) are mast cell activators that provide adjuvant activity when administered by the nasal route. However, small molecule mast cell activators may be a more cost-efficient adjuvant alternative that is easily synthesized with high purity compared to M7 or C48/80. To identify novel mast cell activating compounds that could be evaluated for mucosal vaccine adjuvant activity, we employed high-throughput screening to assess over 55,000 small molecules for mast cell degranulation activity. Fifteen mast cell activating compounds were down-selected to five compounds based on *in vitro* immune activation activities including cytokine production and cellular cytotoxicity, synthesis feasibility, and selection for functional diversity. These small molecule mast cell activators were evaluated for *in vivo* adjuvant activity and induction of protective immunity against West Nile Virus infection in BALB/c mice when combined with West Nile Virus envelope domain III (EDIII) protein in a nasal vaccine. We found that three of the five mast cell activators, ST101036, ST048871, and R529877, evoked high levels of EDIII-specific antibody and conferred comparable levels of protection against WNV challenge. The level of protection provided by these small molecule mast cell activators was comparable to the protection evoked by M7 (67%) but markedly higher than the levels seen with mice immunized with EDIII alone (no adjuvant 33%). Thus, novel small molecule mast cell activators identified by high throughput screening are as efficacious as previously described mast cell activators when used as nasal vaccine adjuvants and represent next-generation mast cell activators for evaluation in mucosal vaccine studies.

## Introduction

Mast cells represent a target for a new class of mucosal vaccine adjuvants. Localized and controlled mast cell activation may be an effective way to induce potent immune responses to co-administered vaccine antigens. Mast cells are innate granulated cells that contain inflammatory mediators pre-stored in their granules (1). Upon activation, mast cells readily release histamine, prostaglandins, leukotrienes, and cytokines (2, 3) that may enhance innate cell migration to immunological inductive sites, such as the draining lymph node, and initiate a host immune response to co-administered vaccine antigens (4–6). Mast cells also synthesize additional inflammatory mediators, such as leukotriene B4, prostaglandin G2, IL-1, IL-3, and IL-10, within hours of activation (7) that may sustain immune responses to increase the potency of vaccine-induced immunity. One special feature of mast cells is the ability to degranulate and re-granulate (2); thus, providing a constant source of localized inflammation readily available to support adjuvant activity. Mast cells contribute to the adjuvant activity of various adjuvants including surfactin, imiquimod, and CTA1-DD/IgG (8–11) although the role of mast cells in the adjuvant activity of some mast cell activators is controversial (12).

Mast cell activators are potent vaccine adjuvants for mucosal delivery. Nasal immunization with Compound 48/80 (C48/80), a cationic polymeric mast cell activating compound (5, 13, 14), combined with a subunit protein antigen induces potent antigen-specific immune responses in mice and rabbits (14–18). Due to the batch-to-batch variability in mast cell degranulation potency of C48/80 (19) and its polymeric nature, C48/80 may provide inconsistent adjuvant activity leading to unacceptable variability in vaccine-induced immunity in the host. Vaccination with mast cell activating peptides, such as Mastoparan 7 (M7) that are synthesized with high purity, may reduce variability in host immune responses. M7 is a highly active analog (20) of the peptide mastoparan and provides potent adjuvant activity after nasal immunization (21, 22). Nasal immunization with M7 and a cocaine-hapten-conjugate vaccine provided effective adjuvant activity and reduced the effects of cocaine-induced locomotion in mice (22). M7 is also a potent mucosal vaccine adjuvant in mice, rabbits, and non-human primates and enhanced antigen-specific immunity when combined with an HIV immunogen (21). Although M7 is an effective mucosal vaccine adjuvant, small molecule mast cell activators may be an additional mast cell activating adjuvant and provide a cost-efficient vaccine adjuvant that would be suitable for future clinical use.

Previously, our group utilized a drug discovery approach and high-throughput screening (HTS) to identify small molecules selected from commercially available compound libraries with mast cell degranulating activity that may be developed as vaccine adjuvants (23). Over 55,000 compounds were evaluated in mouse MC/9 mast cell degranulation assays. Compounds that induced mast cell degranulation in the primary and confirmatory degranulation assays were identified as hit compounds and progressed through the adjuvant discovery pipeline. In the drug discovery literature, a “hit” may be identified as “…a compound which has the desired activity in a compound screen and whose activity is confirmed upon retesting.” (24). Upon identification of hits, additional studies are performed as part of the hit-to-lead phase to identify molecules that are more potent and selective, and suitable for *in vivo* studies (24, 25). After identification of compound leads, reiterative medicinal chemistry to improve the desirable properties of the compounds and formulation studies to improve compound delivery is often performed as a part of lead optimization on the way to developing a clinical candidate drug (24, 25). In the current study, 15 hit small molecule mast cell activators were evaluated for *in vitro* activities in cell types other than mast cells as a part of the hit-to-lead discovery phase. Five lead compounds selected based on diverse *in vitro* activities, functional diversity, and synthesis feasibility, were evaluated for *in vivo* adjuvant activity after nasal delivery using the mouse West Nile Virus (WNV) vaccine as a proof-of-concept model. Three small molecule MCAs enhanced systemic WNV-specific T and B cell responses and induced protection against WNV infection similar to M7. Small molecule MCAs identified by HTS demonstrate adjuvant activity after nasal delivery; but, additional studies are required to optimize the lead compounds’ activities in the adjuvant development phase. The results of the current proof-of-concept study support the use of drug discovery and high throughput screening approaches to identify small molecules MCAs as vaccine adjuvants when delivered by the nasal route.

## Materials and Methods

### Mice

Female BALB/cJ (Jax stock # 000651) and C57BL/6J (Jax stock # 000664) mice (6-8 weeks old) were purchased from the Jackson Laboratory (Bar Harbor, ME). Mice were housed under specific pathogen-free conditions on a twelve-hour light cycle. All experimental procedures were conducted with the approval of Duke University’s Institutional Animal Care and Use Committee.

### Mast Cell Activating Compounds

Small molecule mast cell activating compounds were purchased from TimTec (Newark, DE) or synthesized by Duke University’s small molecule synthesis facility (SMSF). All compounds were prepared in high concentration stocks (20-40 mM) using DMSO (Spectrum Chemical, Gardena, CA) as the solvent for *in vitro* assays and PEG400 (Spectrum Chemical) for *in vivo* assays. Compound solutions were stored at -20°C until used. The mast cell activating peptide mastoparan 7 (M7; amino acid sequence INLKALAALAKALL-NH2) was synthesized by CPC Scientific (San Jose, CA).

### *In Vitro* Compound-Induced Cytokine and Cytotoxicity

Mouse MC/9 mast cells (cat #CRL-8306), JAWSII dendritic cells (cat #CRL-11904), J774A.1 macrophages (cat #TIB-67), and LA-4 lung epithelial cells (cat #CCL-196) were purchased from ATCC (Manassas, VA) and cultured in media according to the instructions provided by ATCC with any modifications described below. MC/9 media was prepared as previously described with the minor modification that used rat T-stim (Cat# 354115 Corning; Corning NY) as a source of growth factors (26). JAWSII cells were cultured in MEM Alpha Modification (HyClone Cat#SH30265.01) with 20% FBS. J774A.1 (27) and LA-4 cells (28) were cultured in media as previously described by others (27, 28). For cytokine induction, 100 μL of cells were plated in 48-well plates at 5 x10^6^ cells/ml in the presence of the 15 hit mast cell activating compounds or M7 at a final concentration of 100 μM. 100 µM was selected based on others using 20 – 200 µM of mast cell activators when tested for mast cell degranulation activity (29). Cells were incubated at 37°C for 24 hours. Supernatants were collected and measured for cytokine content using a 32-cytokine/chemokine multiplex assay from Millipore (Burlington, MA) according to the manufacturer’s instructions. Compound-induced cytotoxicity was also measured 24-hours after stimulation with the mast cell activating compounds tested at 100 μM. Celltiter96™ MTS (Promega; Madison, WI) was added to the cells and incubated for one hour at 37°C before reading absorbance at 490 nm.

### *In Vivo* Mast Cell Activation

BALB/cJ mice were injected subcutaneously with temperature transponders (BMDS; Seaford, DE) one week before *in vivo* mast cell activation evaluation. Baseline temperature was recorded for each mouse before exposure to mast cell activators. Mast cell activating compounds were prepared in a 50% PEG400 solution and M7 was prepared in saline. MCA compounds (20 μmoles) or M7 (200 nmoles) were injected into the mouse peritoneal cavity in 100 μL and evaluated for their ability to activate mast cells as monitored by a drop in body temperature secondary to mast cell degranulation. Temperatures were recorded 15, 30, and 45 minutes after compound exposure. Animals were immediately euthanized by CO_2_ exposure if they displayed a temperature decrease greater than 10°C as a humane endpoint or 45 minutes post-exposure as the experimental endpoint. Change in body temperature was determined by subtracting the baseline temperature from the temperatures recorded 15, 30, and 45 minutes post-exposure.

### *In Vivo* RNA Sequence Analysis

Female C57BL/6J mice (5 mice per group) were nasally instilled with the mast cell activating compounds (200 nmoles), M7 (20 nmoles), MPL (10 μg), or saline in 10 μL. Mice were euthanized six hours post compound exposure. The upper pallet and nasal septum were harvested from each mouse. Harvested tissues were homogenized and total RNA was purified from the homogenized tissues according to the Qiagen^®^ Rneasy kit (Cat.#74106 Hilden, Germany). Total RNA was sequenced utilizing RNA-seq technology performed by BGI^®^ (Cambridge, MA). All genomic analyses used build GRCm38 of the Mus musculus genome. The genome sequence and annotation were downloaded from Ensembl release 98 (30). Analysis was performed using scripts written in the R programming language, Bash, and publicly available software detailed below. Custom Jupyter notebooks, which used the following R and Bioconductor packages: dedexted, DESeq2, EsDb.Mmusculus.v79, foreach, fs, gage, gageData, limma, pathview, pheatmap, plotly, RColorBrewer, Rtse, tidyverse. Basic assessments of sequence data quality were performed using FastQC v0.11.9 (31) and MultiQC v1.9 (32). Raw sequencing reads were trimmed and filtered using fastq-mcf v1.04.807 (33) using the BGISEQ-500 AD1_Long, AD1_Short, AD2_Long, and AD2_Short adapter sequences and their reverse complements (34). Reads were then mapped to the reference genome and read counts were generated using STAR v2.5.4b (35). For quantification of reads mapped to genes, we use the second column of the STAR count output because the libraries were unstranded. Comparative analysis of the resulting count matrices was performed using DESeq2 (36), and the top 20 genes that were under- (negative log-fold change) or over-expressed (positive log-fold change) relative to vehicle only reported for each adjuvant, together with p-values adjusted for multiple comparisons by the Benjamini-Hochberg method (37). Reproducible scripts are maintained under version control at https://gitlab.oit.duke.edu/hy140/staats_adjuvant.

### Mouse Immunization to Evaluate Adjuvant Activity

BALB/cJ mice (n=5 mice per group) received three doses of West Nile Virus envelope domain III (EDIII; GenScript Biotech; Piscataway, NJ) alone (15 μg) or combined with M7 (20 nmoles) or MPL (10 μg) as controls, or the mast cell activating compounds (R127655, R529877, ST101036, ST027688, and ST048871; 200 nmoles) on days 0, 7, and 21 by nasal delivery. For mouse WNV infection studies, BALB/cJ mice (n = 9-13 mice per group) were nasally immunized on days 0, 7, 21, and 35. The first vaccine dose contained 15 µg of EDIII and the subsequent doses contained 30 µg of EDIII on days 7 and 21 alone or co-administered with M7, MPL, or the mast cell activators. Because the small molecule MCAs display hydrophobic properties that were not suitable for co-administration with the aqueous antigen solution, we utilized an immunization method that administered the adjuvant dissolved in a 50%PEG400:water formulation in 15 µL 15-minutes before administering the EDIII antigen in saline (15 µL). On day 35, all vaccine groups were immunized with 30 µg of EDIII alone to boost anti-EDIII antibody responses before a lethal WNV infectious challenge.

### Mouse West Nile Virus Infection

Mice were anesthetized with isoflurane. West Nile Virus, strain NY99-35262-11 (BEI Resources NR-677) was diluted to 1.6 x10^5^ PFU/mL. Each mouse was injected with 0.1 mL of the virus *via* the intraperitoneal route. After mice fully recovered from anesthesia, the animals were returned to their home cages and provided water and chow *ad libitum*. Changes in body weight, temperature, and activity were monitored daily for 14 days after challenge. Animals that displayed a 20% weight loss versus pre-challenge weight or developed hind limb paralysis were humanely euthanized (38). All work with live virus and infected animals was completed in an animal biosafety level (ABSL)-3 suite in the Regional Biocontainment Laboratory (RBL) at Duke University.

### Serum Collection for ELISAs

Blood was obtained from immunized mice using the submandibular lancet method. The whole blood was centrifuged for 10 minutes at 4°C. Serum was collected by removing the supernatant from the clotted blood and stored at -20°C until analysis.

### ELISA

ELISAs were performed as previously described (20) with modifications indicated below. 384-well black plates (Cat. # 460518 Thermo Scientific; Watham, MA) were coated with WNV EDIII as the coating antigen (2 µg/ml) diluted in carbonate/bicarbonate buffer (CBC, pH 9.5). Serum was diluted two-fold beginning at 1:32 in sample diluent (PBS, 1% BSA, 1% NFDM, 5% goat serum, 0.05% Tween 20, and 0.1% 2-Chloroacetamide). Goat anti-mouse IgG, IgG1, and IgG2a, alkaline phosphatase-conjugated antibodies (Southern Biotech; Birmingham, AL) were diluted 1:8,000 in secondary antibody diluent (PBS, 1% BSA, 5% goat serum, 0.05% Tween 20, and 0.1% 2-Chloroacetamide). The fluorescent Attophos substrate (Promega; Madison, WI) was added to each well and incubated for 15 minutes before reading plates in a BioTek Synergy 2 plate reader using 440/30 nm excitation and 560/40 nm emission filters to detect the light signal produced by the Attophos substrate after enzymatic activation by the alkaline-phosphatase-conjugated detection antibodies. The fluorescent signal was reported as relative light units (RLU). Endpoint titers, defined as the last log2 immune sample dilution with an RLU signal 3-fold greater than a naïve reference sample at the same dilution, were used for statistical analysis. Graphs presented in the figures were prepared using geometric mean titer antilog values.

### Splenocyte Antigen Restimulation Cytokine Assay

BALB/cJ mice (5 mice per group) in the immunogenicity study were euthanized three weeks after the final immunization and spleens from each mouse were harvested and processed into single-cell suspensions. Single-cell suspensions were prepared from whole spleens by cutting each spleen into small pieces followed by pressing through a 70 µM filter using the rubber end of a sterile 3 ml syringe plunger. Cells were pelleted by centrifugation and red blood cells were lysed using RBL Lysis Buffer (Cat# R7757 Sigma; St. Louis, MO) according to the manufacturer’s recommendation. Splenocytes were washed twice in RPMI-1640 containing 5% FBS (Cat#25-514 Genesee; San Diego, CA) and 1% penicillin/streptomycin (Cat#15140122 ThermoFisher; Waltham, MA) before counting and plating. Splenocytes were plated in 48-well plates (2.5 x 10^6^ cells/well) and cultured in media alone (39) or media containing EDIII (25 μg/ml) in a total volume of 500 µl for 72 hours at 37°C and 5% CO_2_. Supernatants were collected and measured for IL-4, IL-5, IL-17, and IFN-γ using the Bioplex Multiplex (Biorad; Hercules, CA) according to the manufacturer’s instructions. EDIII-induced cytokine responses were determined by subtracting the cytokine value from cells cultured in media from the cytokine values measured in cells stimulated with EDIII.

### Flow Cytometry to Monitor *In Vivo* Cellular Infiltration

C57BL/6J mice (5 mice per group) were nasally instilled with one of the five lead mast cell activating compounds (200 nmoles) or M7 (20 nmoles). Twenty-four hours after compound exposure, draining cervical lymph nodes were harvested from mice and digested with a digestion buffer containing 10% collagenase [Cat. #C2674, Sigma (St. Louis, MO)], 1% deoxyribonuclease I [Cat. #04716728001, Roche (Basel, Switzerland)], 10 mM HEPES, and 1.5% FBS in HBSS to make single-cell suspensions. Cells were washed and pre-stained with Live/dead staining solution (Zombie Violet™ Fixable Viability Kit, Biolegend) for 10 minutes at room temperature. After washing, cells were stained with fluorescently labeled antibodies against B220 (clone RA3-6B2), CD11b (clone M1/70), CD11c (clone N418), CD45 (clone 30-F11), CD86 (clone GL01), IA/IE (clone M5/114.15.2), and isotype controls (BioLegend, San Diego, CA) for 30 minutes at 4°C. To examine the expression levels of individual markers, an LSR II flow cytometer (BD; Franklin Lakes, NJ) was utilized to analyze the samples. Data analysis was performed using FlowJo software (Tree Star, Ashland, OR).

### Statistical Analyses

GraphPad Prism Version 9 (San Diego, CA) was used to identify statistical differences between groups in each experiment. One-way analysis of variance (ANOVA) determined if mast cell activating compounds or the positive controls statistically enhanced responses in the *in vivo* mast cell degranulation, *in vivo* cellular infiltrate, and *in vivo* immunogenicity assays using the Dunnett’s multiple comparison test compared to the negative controls. In the WNV infection study, one-way ANOVA determined if any adjuvant provided superior adjuvant activity by comparing antibody responses induced by each adjuvant to the other adjuvants using Tukey’s multiple comparison post-test. Survival curves were analyzed using the Gehan-Breslow-Wilcoxon test *p < 0.05, **p < 0.01, ***p < 0.001, and ****p < 0.0001.

## Results

### Compounds That Activate Mast Cells Also Induce Cytokine Production in Other Cell Types

**Figure 1:**
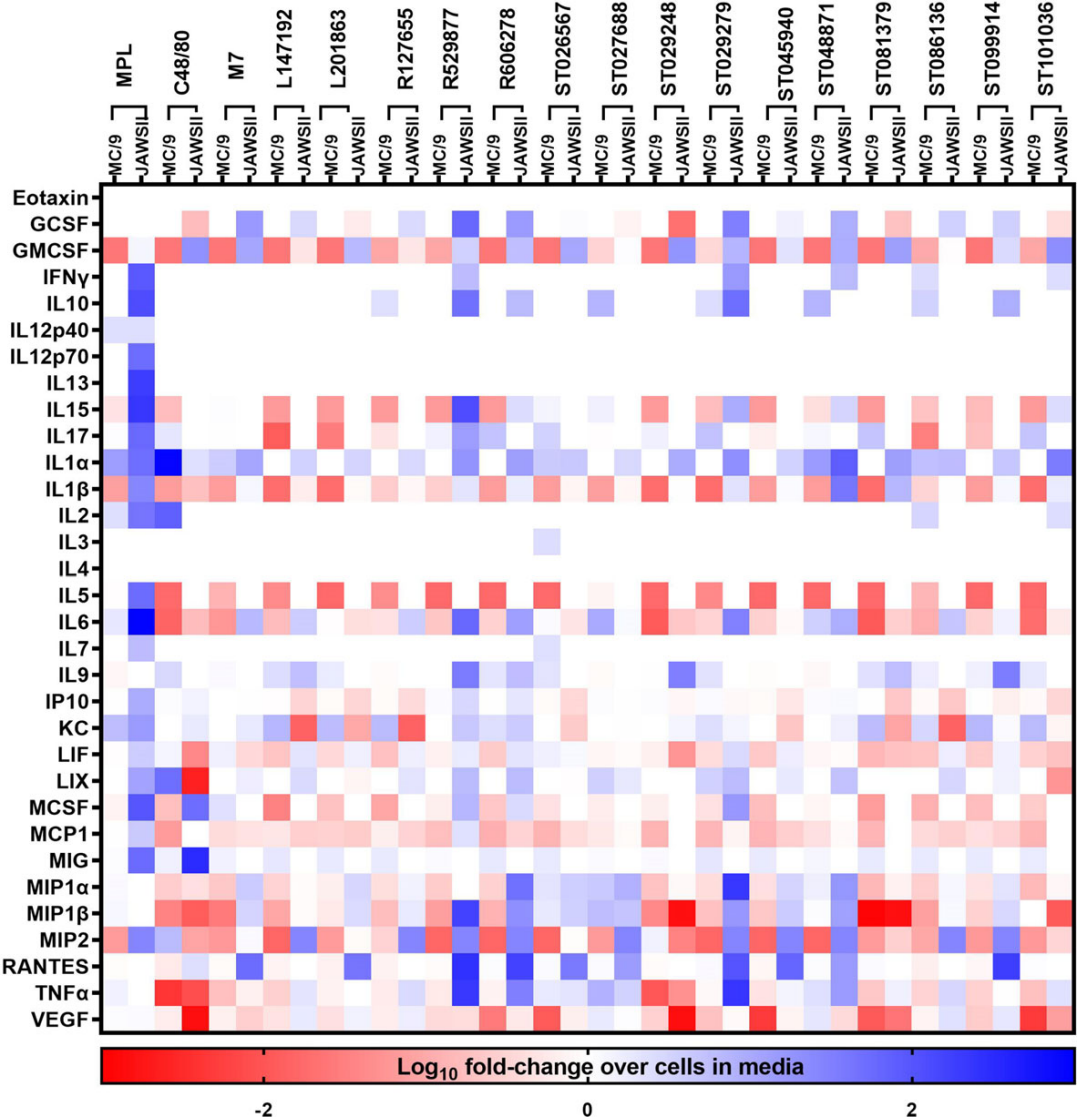
Small molecule mast cell activators activate mouse innate immune cells. Mouse MC/9 mast cells or JAWSII dendritic cells were incubated in the presence of the small molecule mast cell activators (100 μM) or positive control vaccine adjuvants MPL, C48/80, or M7 for 24 hours. Cell supernatants were analyzed for cytokine production using a mouse 32-plex multiplex assay. Cytokine production was calculated as a fold-increase compared to cells cultured in media without additional stimuli. Data represents the log10 of the fold-increase in cytokine response.

Fifteen hit small molecule mast cell activators identified from our previous high-throughput screening of commercially available compound libraries to discover small molecules that induce mast cell degranulation (22) were evaluated for acute production of inflammatory mediators from mast cells. Similarly, compounds that induce cytokine and chemokine production in mast cells, may also activate other cells to produce inflammatory mediators to contribute to the adjuvant activity. Therefore, compound-induced cytokine and chemokine responses were evaluated in mouse mast cells (MC/9) and dendritic cells (JAWSII) (**Figure 1**) to determine the ability of compounds that induce immediate mast cell degranulation to activate other innate immune cells. Mouse mast cells and dendritic cells were stimulated with the 15 hit MCA identified from our primary screen (22) or C48/80, M7, or MPL as positive controls with known vaccine adjuvant activity for 24 hours and monitored for cytokine production using a 32-panel cytokine/chemokine multiplex assay. JAWSII cells were more responsive to MPL than MC/9 cells and MPL enhanced the secretion of several cytokines including IFN-γ (90-fold), IL-1α (49-fold), IL-1β (26-fold), IL-6 (942-fold), and MCSF (96-fold) when compared to levels produced in MC/9 cells. Conversely, C48/80 induced a stronger cytokine response in MC/9 cells than JAWSII cells as indicated by enhanced IL-1α (871-fold), IL-2 (82-fold), LIX (142-fold), and MIP2 (7-fold) production. M7 induced a similar cytokine response in MC/9 cells as C48/80 with enhanced IL-1α (655-fold), IL-2 (51-fold), LIX (92-fold), and MIP2 (5-fold) suggesting known MCA induce similar cytokine responses in mast cells. While MPL induced MC/9 cells to secrete IL-1α (100-fold) and IL-2 (10-fold) it was to a lesser extent than C48/80 or M7. IL-1α was secreted in response to the small molecule MCAs in several cell types including MC/9, JAWSII (**Figure 1**), macrophages (J774A.1), and lung epithelial cells (LA-4) (**Supplementary Tables 1–4**). ST026567 (78-fold), ST086136 (208-fold), and ST048871 (117-fold) enhanced IL-1α production in MC/9 cells and ST048871 (78-fold) and ST101036 (34-fold) were the most potent inducers of IL-1α in JAWSII cells. Several small molecule MCAs induced KC responses in MC/9 cells similar to MPL including, L147192, L201863, R127655, ST081379, ST086136, ST099914, and ST101036. However, the MCAs did not induce similar KC responses as MPL in JAWSII cells. Some compounds such as ST027688, ST026567, and ST086136 induce MC/9 cells to enhance secretion of several cytokines whereas other compounds including R529877 and ST045940 do not induce MC/9 cells to secrete any cytokines under the conditions tested described in this study. Conversely, R592877 induces a potent cytokine response in JAWSII cells including GCSF (60-fold), IL-15 (130-fold), IL-6 (62-fold), MIP-1α (1762-fold) and MIP-1β (428-fold), and TNF-α (2071-fold). Similarly, several compounds that induce mast cell activation including, R606278, ST029279, and ST048871 also enhance cytokine secretion in JAWSII cells, suggesting the ability of compounds that activate mast cells to also activate other innate immune cells.

### Mast Cell Activating Compounds Exhibit Variable Cytotoxicity in Innate Immune Cells

**Figure 2:**
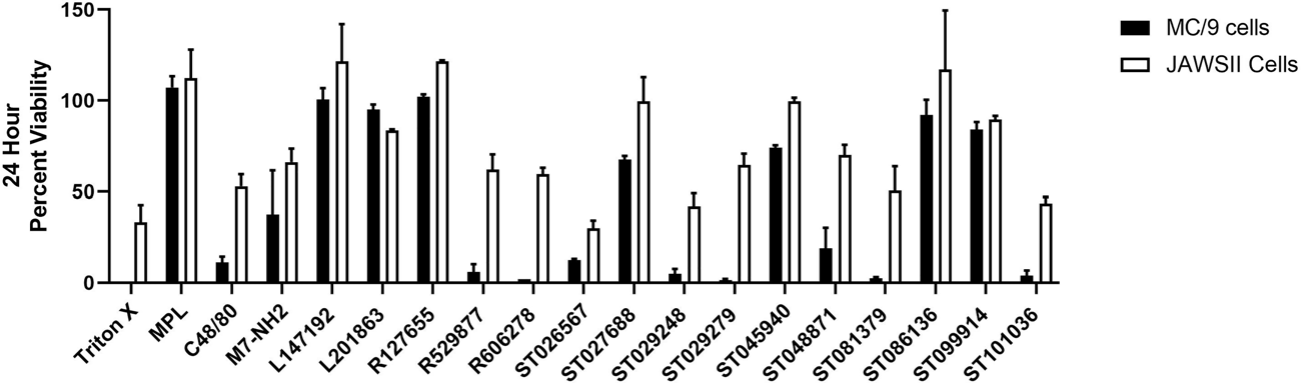
Small molecule mast cell activators induce unique cytotoxicity profiles in mouse innate immune cells. Mouse MC/9 mast cells or JAWSII dendritic cells were incubated in the presence of the small molecule mast cell activators (100 μM) or positive control vaccine adjuvants MPL, C48/80, or M7 for 24 hours. Cell viability was measured using a colorimetric MTS assay.

Cytokine production can be influenced by cell viability. Therefore, we monitored the viability of mouse MC/9 cells and JAWSII cells after exposure to the MCAs for twenty-four hours (**Figure 2**), which corresponds to the timepoint for cytokine/chemokine analysis (**Figure 1**). MC/9 cells were more susceptible to cell death than JAWSII cells. MPL did not induce cytotoxic effects in either cell line; however, both C48/80 and M7 reduced cell viability in MC/9 (11% and 37% viable, respectively) and JAWSII (53% and 66% viable, respectively) cells. Compounds R529877 (6% viable), R606278 (2% viable), ST026567 (12.5% viable) ST029248 (5% viable), ST029279 (1.5% viable), ST081379 (2.5% viable) and ST101036 (4% viable) were highly cytotoxic in MC/9 cells. Conversely, ST026567 (30% viable), ST029248 (42% viable) and ST101036 (43.5% viable) were the most cytotoxic compounds in JAWSII cells and reduced cell viability to less than 50%. However, because some compounds display cytotoxicity and enhance cytokine production such as ST026567 and ST029279 in MC/9 cells and ST026567 and ST101036 in JAWSII cells, it is not clear how cell viability influences compound-induced cytokine production. Thus, cytokine production and cellular cytotoxicity may be independent measurements for some mast cell activators that should be considered when selecting small molecule mast cell activators for evaluation as *in vivo* vaccine adjuvants.

### Small Molecule MCAs Induce Mast Cell Degranulation *In Vivo*

**Figure 3:**
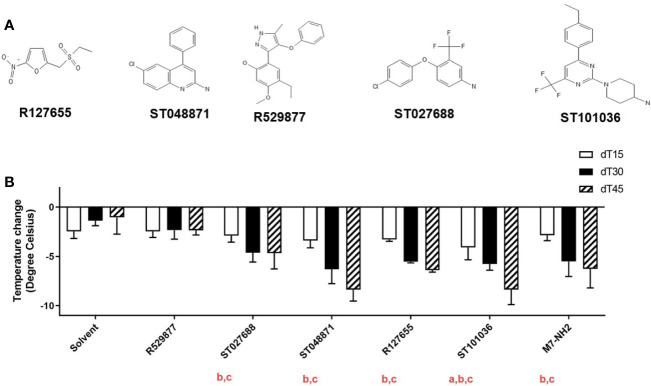
Small molecule mast cell activators induce mast cell activation after intraperitoneal injection. Five mast cell activating compounds **(A)** (20 μmoles) or M7-NH2 (200 nmoles) were injected in the peritoneal cavity of naïve BALB/c mice (n= 3-12 mice per group) to monitor for mast cell activation *via* hypothermia secondary to mast cell degranulation **(B)**. Temperatures were recorded before compound exposure and every 15 minutes after exposure for 45 minutes. Change in body temperature was determined by subtracting pre-exposure temperature from the temperatures recorded 15, 30, and 45 minutes after compound injection. One-way ANOVA determined if compounds induced a significant decrease in core body temperature compared to solvent at a: 15 minutes, b: 30 minutes, and c: 45 minutes after exposure. Bars represent the mean + SD.

The fifteen small molecule MCAs hits were reduced to five lead MCAs for *in vivo* evaluation. Compounds R127655, R529877, ST027688, ST048871, and ST101036 (**Figure 3A**) were selected for *in vivo* evaluation based on their diverse *in vitro* activities and synthesis feasibility. R127655 is a potent activator of lung epithelial cells and R529877 is a potent dendritic cell activator. ST027688 is a strong MC/9 activator for cytokine production and ST048871 strongly activates MC/9 cells and J774A.1 cells. ST101036 was selected based on its potent MC/9 degranulation activity and its ability to induce cytotoxicity in MC/9, LA-4, J774A.1, and JAWSII cells, similar to C48/80. These five lead compounds were screened for *in vivo* activity using a mouse model of compound-induced hypothermia. Immediate hypothermia is used as an indicator of mast cell activation because systemic mast cell degranulation often results in anaphylactic symptoms and can be observed by a decrease in body temperature (40). MCAs were dissolved in a vehicle solution containing PEG400 and saline (solvent) before injection into the mouse peritoneal cavity at a dose of 20 μmoles. Core body temperatures for each mouse were measured before injecting the compound and every 15 minutes after injection for 45 minutes (**Figure 3B**). The solvent alone caused a temperature decrease ranging from -1.3 to -2.2°C over the 45-minute duration of the experiment. R529877 (-2.5°C) did not induce hypothermia different than the vehicle solvent. However, ST027688 (-4.7°C), ST048871 (-5.9°C), and R127655 (-5.5°C) and M7 (-5.2°C) induced hypothermia greater than the solvent 30 minutes after exposure and maintained a hypothermic state at 45 minutes post-exposure. The MCA ST101036 induced the strongest decrease in body temperature 15 minutes after exposure (-4.2°C), which was maintained at 30 (-5.4°C) and 45 minutes (-9.3°C) post-exposure. Four of the five small molecule MCAs induce mast cell activation *in vivo*, comparable or greater than M7, as determined by compound-induced hypothermia.

### Small Molecule MCAs Alter NALT Gene Expression After Nasal Immunization

**Figure 4:**
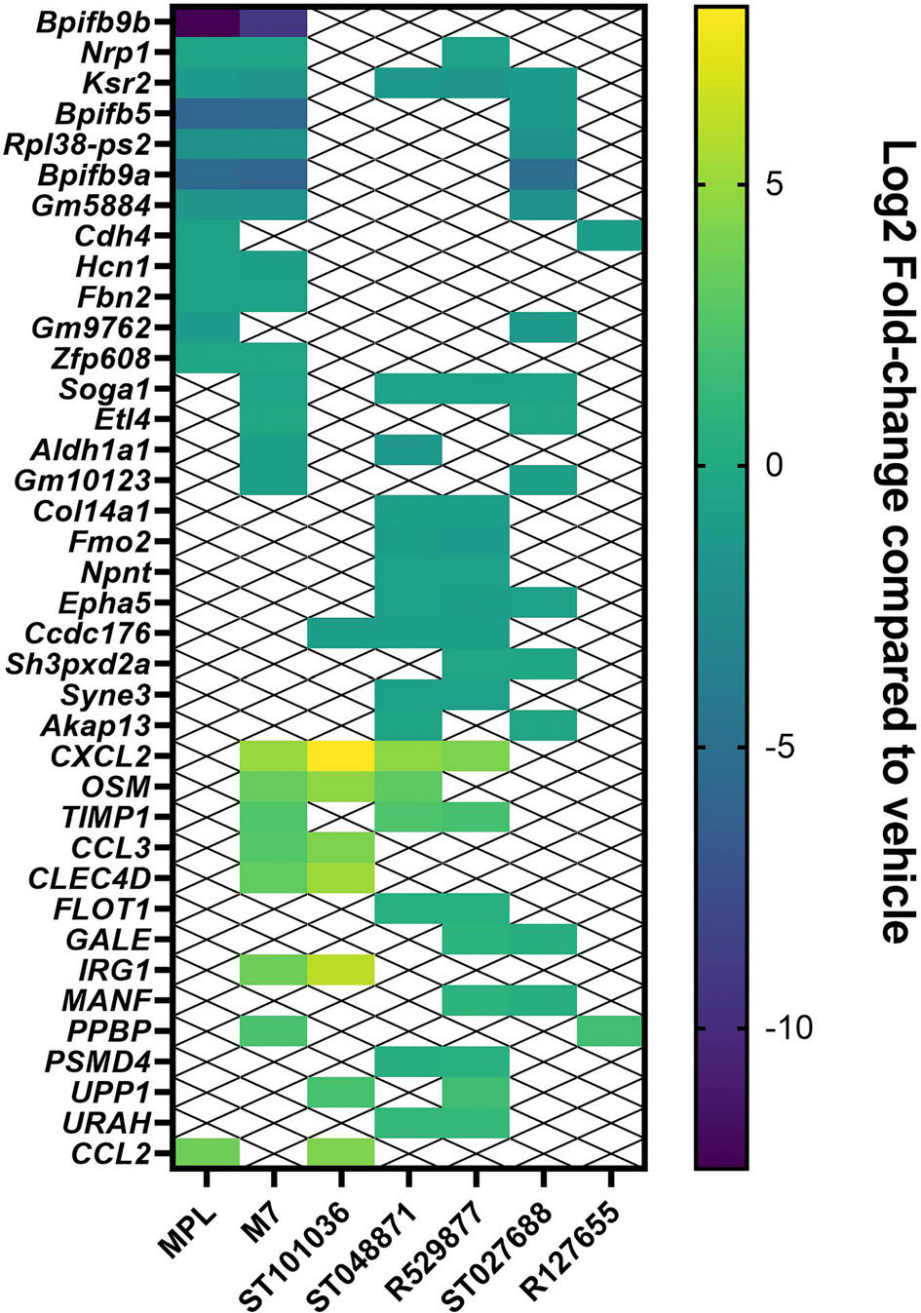
Small molecule mast cell activators modify gene expression in the NALT after nasal delivery. Lead small molecule mast cell activators, M7 and MPL were nasally administered to C57BL/6 mice (n = 5 mice per group). NALT cells were harvested from each mouse six hours after compound delivery and processed for RNA sequencing. Genes enhanced or inhibited by more than one compound were compared amongst vaccine adjuvants. The Log2 fold-change over vehicle control for each gene is presented.

Our previous results with a variety of nasal vaccine adjuvants, including the mast cell activator C48/80, suggests nasal exposure to vaccine adjuvants may activate the innate immune system to produce local cytokine secretion in the nasal cavity within a few hours after delivery (17, 41). We, therefore, evaluated MCA-induced gene expression changes in the nasal-associated lymphoid tissue (NALT) as an unbiased method to evaluate *in vivo* activity of the small molecule MCA at the site of vaccination. The five lead MCAs, R127655, R529877, ST027688, ST048871, and ST101036, vehicle negative control, or positive control vaccine adjuvants M7 and MPL were administered to mice by the nasal route. Six hours after nasal delivery of compounds, the NALTs were collected for gene expression analysis. The top 20 genes enhanced (**Supplementary Table 5**) or inhibited (**Supplementary Table 6**) each MCA or control adjuvants and enhanced or inhibited by multiple adjuvants (**Figure 4**) were compared to changes in gene expression induced by the vehicle control. All small molecule MCA induced measurable changes in gene expression after nasal delivery to mice. R529877, ST048871, and ST101036 enhance the expression of several genes shared amongst other MCAs, including M7. *Ccl3* and *cxcl2* are associated with chemotactic immune responses. M7 and ST101036 enhanced ccl3 expression and M7, R592877, ST048871, and ST101036 enhanced *cxcl2* expression. M7 and ST101036 both enhance expression of *Clec4d* [C-type lectin receptor CLECSF8 (CLEC4D)] (42) and *Irg1* (Immune-Responsive Gene 1*)* and thus may be associated with activation of innate immune cells. *Osm* (oncostatin M) is a gene that regulates cytokine production (43), including IL-6, G-CSF, and GM-CSF, and is enhanced after exposure to ST101036, ST048871, and M7. Although ST027688 and R127655 also enhance gene expression in the NALT, they enhance fewer genes that are shared between other MCAs. R127655 and ST027688 enhance one and two genes, respectively, that are shared with other MCAs, including *ppbp* (pro-platelet basic protein, R127655), which is the gene encoding for the chemokine *cxcl7* (44) and *gale* (UDP-galactose 4’-epimerase, ST027688), which is an enzyme that catalyzes galactose metabolism (45), and *manf* (Mesencephalic astrocyte-derived neurotrophic factor, ST027688), which influences macrophages to achieve an anti-inflammatory phenotype (46). We interpret the observation of several genes being enhanced by more than one MCA, including the positive control M7, to indicate that the small molecule MCAs are activating similar pathways after nasal delivery to mice. In contrast to genes that were enhanced by more than one MCA, only one gene, *ccl2*, was enhanced by MPL and an MCA (ST101036). Although MPL also induced the expression of *cxcl10*, *irf7*, and *ifit3*, these genes were not induced by any of the MCA, suggesting that nasal delivery of MCA activates the innate immune system *via* a pathway that is largely different from that activated by MPL.

RNA sequence analysis also identified genes that were inhibited by multiple compounds after nasal delivery of the small molecule MCAs or the controls M7 and MPL (**Figure 4**). Gene expression inhibited after nasal exposure to the small molecule MCAs and control adjuvants appear to be associated with structural proteins and smell sensory. *Bpifb5* and *Bpifb9a*, which are associated with lipid binding, perception of smell and are bactericidal genes found in olfactory tissues (47), were inhibited by MPL, M7, and ST027688 suggesting these compounds and control adjuvants share common pathways for gene inhibition. Similarly, MPL, M7, and MCAs ST048871, ST027688, and R529877 all inhibited *ksr2* (kinase suppressor of Ras 2), which assists with energy regulation (48). R127655 and ST101036 have the least inhibited genes in common with other MCAs and MPL. R127655 and ST101036 only share one inhibited gene, *cdh4*, and *ccdc176*, respectively, in common with another MCA or control adjuvant. R529877, ST048871, and ST101036 inhibit *Ccdc176* expression and MPL and R127655 inhibit *Cdh4* expression. *Ccdc176* (49) and *Cdh4* (50) are genes involved in maintaining structural integrity including, cilia orientation and cell adhesion. Despite observing unique enhanced gene expression profiles between MPL and the MCA (including M7), which may indicate activation of diverse innate immune pathways; shared inhibited gene expression profiles amongst MPL and the MCA (including M7) suggests nasal delivery of vaccine adjuvants downregulates genes in a more generalized response that is less specific than the mechanisms that lead to enhanced gene expression. Although more studies are needed with additional sample collection time points to fully understand gene expression changes induced by the small molecule MCAs and control adjuvants MPL and M7, our RNA sequencing results combined with cytokine production in mouse mast cells, dendritic cells (**Figure 1**), macrophages, and lung epithelial cells (**Supplementary Tables 2**, **3**) suggests the small molecule MCAs activate components of the innate immune system.

### Small Molecule MCAs Provide Nasal Adjuvant Activity in Mice After Administration With West Nile Virus Subunit Vaccine

**Figure 5:**
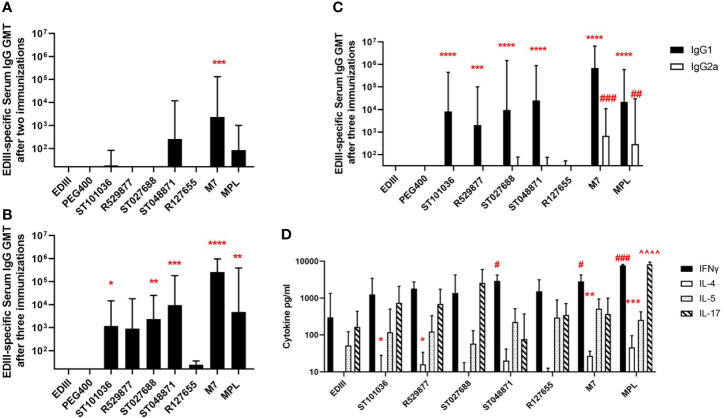
Small molecule mast cell activators provide nasal adjuvant activity when co-administered with WNV EDIII. Female BALB/c mice (n = 5 mice per group) were nasally immunized with EDIII alone in saline or PEG400 or combined with MCAs, MPL, or M7. Vaccines were administered on days 0, 7, and 21. Serum collected after two immunizations were measured for EDIII-specific IgG **(A)**, while serum collected after three immunizations was measured for EDIII-specific IgG **(B)**, IgG1 (*), and IgG2a (#) **(C)**. EDIII-induced cytokine responses were measured in splenocytes after immunization [statistical indicators for each cytokine; IL-4 (*), IFN-γ (#), and IL-17 (^)] **(D)**. One-way ANOVA was used to determine if adjuvants statistically increased EDIII-specific antibody responses compared to mice immunized with EDIII in saline. *p < 0.05, **p < 0.01, ***p < 0.001, ****p < 0.0001. Antibody responses represent the geometric mean titer + geometric SD. Cytokine (pg/ml) responses are presented as mean + SD. The ^#^ denotes significant differences in IFN-g responses and ^ denotes significant differences in IL-17 responses. The number of ^#^ or ^ denotes the range of the p-value. ^##^p < 0.01 IFN-g, ^###^p < 0.001 IFN-g, and ^####^p < 0.0001 IFN-g. ^^^^p < 0.0001 IL-17.

Because MCAs possessed *in vivo* activity in the mast cell activation and RNA sequence expression assays, we next evaluated the five lead MCAs for *in vivo* adjuvant activity using a mouse model of West Nile virus (WNV) envelope domain III (EDIII) nasal vaccination. BALB/cJ mice received three nasal immunizations with the EDIII antigen (15 µg) alone or combined with one of the five lead MCAs (200 nmole dose) (R127655, R529877, ST027688, ST048871, ST101036), or M7 (20 nmoles) or MPL (10 µg) as positive control adjuvants. Due to the hydrophobic nature of the MCAs, a vehicle control, containing antigen in PEG400 (the diluent used for the MCA) was also included. ST048871 (1: 2.6 x10^2^ GMT) was the only MCA to induce detectable EDIII-specific serum IgG antibodies after two immunizations and M7 (1: 2.4 x10^3^ GMT, p =0.0006) was the only adjuvant that increased EDIII-specific serum IgG after two nasal immunizations when compared to the anti-EDIII IgG response in mice immunized with EDIII alone (**Figure 5A**). However, after three immunizations, ST101036 (1: 1.2 x10^3^ GMT, p =0.03), ST027688 (1: 2.3 x10^3^ GMT, p = 0.009), ST048871 (1: 9.4 x10^3^ GMT, p = 0.0005), M7 (1: 2.6 x10^5^ GMT, p < 0.001) and MPL (1: 4.7 x10^3^ GMT, p = 0.002) statistically increased EDIII-specific serum IgG when compared to response in mice immunized with EDIII alone (1:16 GMT) (**Figure 5B**). R529877 (1: 8.9 x10^2^ GMT, p = 0.053), R127655 (1:24 GMT) and EDIII in PEG400 (1:16 GMT) did not induce EDIII-specific IgG responses different from EDIII alone in saline. The MCAs induce a dominant IgG1 antibody response and four of the five MCAs (R529877, ST027688, ST048871, and ST101036) increased EDIII-specific IgG1 compared to mice immunized with EDIII alone. However, only M7 (1: 6.8 x10^2^ GMT, p = 0.0005) and MPL (1: 3.0 x10^2^ GMT, p = 0.009) induced enhanced EDIII-specific IgG2a when compared to mice immunized with EDIII alone (**Figure 5C**). Our results demonstrate four mast cell activating small molecules, R529877, ST027688, ST048871, and ST101036, provided nasal vaccine adjuvant activity as indicated by their ability to induce elevated EDIII-specific serum IgG responses.

Antigen-specific splenocyte cytokine responses were monitored to characterize the adjuvant-induced T cell responses in mice nasally-immunized with WNV EDIII +/- MCA, M7, or MPL (**Figure 5D**). Cytokines monitored were selected based on their association with Th17 (IL-17), Th1 (IFNγ), and Th2 (IL-4, IL-5) responses. IL-17 and IFNγ were the dominant cytokines produced by spleen cells from immunized mice after stimulation with EDIII. MPL induced the strongest IFNγ (7754 pg/ml, p = 0.0004) response compared to mice immunized with EDIII alone. ST048871 was the most potent MCA for IFNγ production (2933 pg/ml, p = 0.02), which was similar to M7 (2840 pg/ml, p = 0.03). R127655, R529877, ST101036, and ST027688 induced similar levels of IFNγ (1367-1802 pg/ml) and were not statistically different than the IFNγ production observed in splenocytes from mice immunized with EDIII alone (300 pg/ml). ST027688 (2580 pg/ml; p = 0.084) induced elevated IL-17 responses; however, MPL (8233 pg/ml, p < 0.0001) was the only adjuvant to increase IL-17 production greater than EDIII alone (165 pg/ml). The amount of IL-4 induced by EDIII was relatively low but R529877 (16.1 pg/ml, p = 0.02), ST101036 (8.2 pg/ml, p = 0.02), M7 (27.2 pg/ml, p = 0.007) and MPL (46 pg/ml, p = 0.0006) statistically enhanced IL-4 responses compared to EDIII alone (undetectable). There were no observable differences in adjuvant-mediated IL-5 responses. MCA small molecules provide nasal adjuvant activity that enhances antigen-specific IL-4 (R529877 and ST101036) and IFNγ (ST048871) responses after nasal immunization.

### Small Molecule MCAs Enhance Protective Immunity Against West Nile Virus Infection

**Figure 6:**
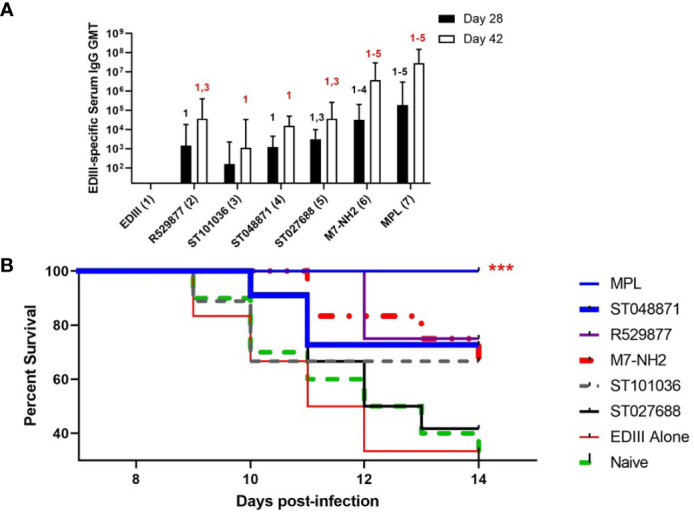
Small molecule mast cell activators provide nasal adjuvant activity when co-administered with WNV EDIII and enhance survival after the WNV challenge. Female BALB/c mice were nasally immunized with EDIII alone (n=12) or combined with R529877 (n=12), ST101036 (n=9), ST048871 (n=11), ST027688 (n=12), M7 (n=12), or MPL (n=13). Vaccines were administered on days 0, 7, and 21 with antigen combined with adjuvant and again on day 35 with antigen only. Serum collected after three and four vaccine doses was measured for EDIII-specific IgG **(A)**. One-way ANOVA with Tukey’s multiple comparisons was used to determine if any vaccine formulation statistically increased EDIII-specific antibody responses compared to the other vaccines tested. The numbers above each bar indicate a statistical increase in antibody response compared to the corresponding vaccine group. Antibody titer bars represent the geometric mean titer + geometric SD. After immunization, mice were infected ***p < 0.001 compared to mice immunized with EDIII alone. with WNV and monitored for survival for 14 days after infection **(B)**.

After detecting enhanced antigen-specific humoral and cellular immune responses in mice nasally immunized with WNV EDIII combined with four of the five lead MCAs (R529877, ST027688, ST048871, and ST101036) we next determined if any MCA compounds provided nasal vaccine adjuvant activity that protects against WNV infection. BALB/cJ mice (9-13 per group) were immunized on days 0, 7, and 21 with EDIII alone or combined with ST048871, ST027688, R529877, ST101036, M7, or MPL. R127655 was excluded from this study because it did not enhance antigen-specific serum antibody or T cell cytokine responses in previous nasal immunization studies. Because the goal of this experiment is to determine the ability of MCA adjuvants to induce protective immunity against a WNV infection we administered an additional vaccine dose on day 35 that contained EDIII alone to boost EDIII-specific antibody responses before the evaluation of protective immunity. Serum collected after three nasal vaccinations (Day 28) indicated that ST101036 (1:161 GMT, p = 0.12) was the only MCA that did not enhance EDIII-specific IgG compared to the EDIII-specific IgG response observed in mice immunized with EDIII alone (1:16 GMT). ST027688 (1: 3.1 x 10^3^ GMT, p = 0.02) induced EDIII-specific IgG antibodies superior to ST101036 after three doses. M7 (1: 3.3 x 10^4^ GMT) induced EDIII-specific IgG responses that were elevated compared to those induced in mice immunized with EDIII alone (p < 0.0001), or mice immunized with EDIII combined with R529877 (1:1.4 x 10^3^ GMT, p = 0.004), ST101036 (p < 0.0001) or ST048871 (1: 1.2 x 10^3^ GMT, p = 0.003). MPL (1: 1.9 x 10^5^ GMT, p < 0.0001) induced EDIII-specific IgG antibodies that were enhanced compared to all of the small molecule MCAs after three vaccine doses (**Figure 6A**). The addition of a fourth vaccine dose containing EDIII only boosted EDIII-specific IgG responses in adjuvanted vaccine groups measured on Day 42. All vaccine adjuvants tested enhanced EDIII-specific serum IgG responses when compared to EDIII-specific IgG results observed in mice immunized with EDIII alone (1:16 GMT) after four vaccine doses. ST101036 (1: 1.1 x 10^3^ GMT, p = 0.0001) and ST048871 (1: 1.5 x 10^4^ GMT, p < 0.0001) were the least potent MCAs and enhanced EDIII-specific IgG compared to EDIII alone. R529877 (1: 3.7 x 10^4^ GMT, p = 0.003) and ST027688 (1: 3.7 x 10^4^ GMT, p = 0.003) increased EDIII-specific IgG compared to ST101036. M7 (1: 3.7 x 10^6^ GMT, p< 0.0001) and MPL (1: 2.9 x 10^7^ GMT, p < 0.0001) were the most potent nasal vaccine adjuvants and induced EDIII-specific IgG that was elevated compared to EDIII-specific IgG responses observed in mice immunized with EDIII and the MCA small molecules. Two weeks after the last immunization, immunized mice and unimmunized naïve mice were infected with WNV NY99 and monitored for survival for 14 days (**Figure 6B**). Thirty percent of naïve mice and 33% of mice immunized with EDIII alone survived WNV exposure. ST027688 was the least effective adjuvant for protecting against WNV as only 42% of ST027688 exposed mice survived WNV exposure. ST101036 (p=0.33) protected 67% of mice exposed to WNV, which is similar to M7 (p=0.033). R529877 (p=0.012) and ST048871 (p=0.072) were the most effective MCA small molecules and protected 75% and 73% of mice, respectively, from WNV. MPL (p= 0.0005) was the only adjuvant to induce 100% protection against morbidity after WNV challenge. Our results demonstrate that small molecule MCAs provide adjuvant activity for nasally-administered vaccines and provide measurable protection against an infectious viral challenge. While the small molecule MCAs did not provide 100% protection against virus-induced morbidity, the results from these *in vivo* studies represent the first *in vivo* studies to evaluate the nasal adjuvant activity of small molecule MCA identified from commercially available compound libraries.

### MCAs M7, ST101036, and R592877 Recruit B220+ Cells to the Draining Cervical Lymph Nodes After Nasal Vaccination

**Figure 7:**
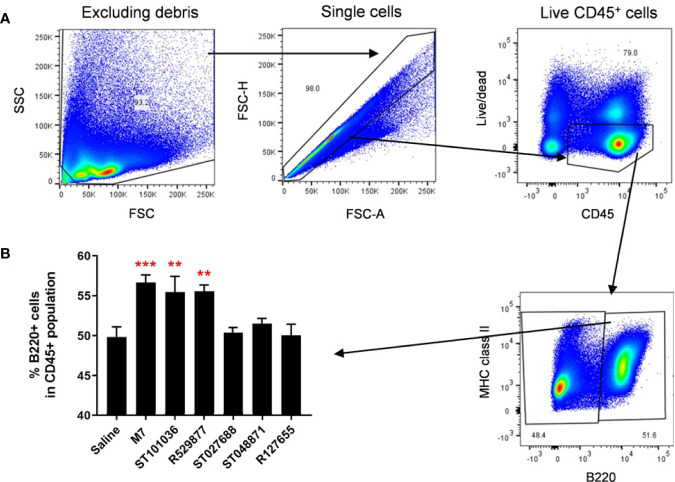
Mast cell activators increase B220+ cells in the cervical lymph nodes after nasal exposure. C57BL/6 mice were nasally instilled with saline, M7, ST101036, R127655, R529877, ST027688, or ST048871. After 24 hours, cervical lymph nodes (cLN) were obtained. Single-cell suspension of isolated cLNs was analyzed by flow cytometry **(A)**. One-way ANOVA determined a significant increase compared to saline **p < 0.01, ***p < 0.001. Bars represent the mean + SEM **(B)**.

The five lead small molecule MCAs were evaluated for their ability to influence the recruitment of antigen presenting cells into the cervical lymph node because the CLN represents a lymphoid inductive site that drains the upper respiratory tract in mice (51). M7, MCA small molecules, or saline were nasally delivered to mice. Twenty-four hours later, CLNs cells were monitored for CD11c (classical dendritic cells, cDC) and B220 (plasmacytoid dendritic cells (pDC) and B cells) (52, 53) expression by flow cytometry (**Figure 7A**). A difference in CD11c+ cell populations was not observed after exposure to saline or the vaccine adjuvants (data not shown). However, ST101036 (p =0.006), R529877 (p = 0.005), and M7 (p = 0.0007) increased the percentage of B220+ cells in the CLN compared to saline (**Figure 7B**). Both ST101036 and R592877 small molecules and the M7 peptide provided adjuvant activity and increased the percentage of B220+ cells in the CLN after nasal delivery. Although the small molecule ST048871 provided effective nasal adjuvant activity (**Figure 6**), its use as a nasal vaccine adjuvant did not result in an increase in the percentage of B220+ cells in the CLN after nasal delivery. Our results suggest recruitment of B220+ cells may be observed in the CLN after nasal delivery of small molecule MCA but it is not required for nasal adjuvant activity of small molecule MCA.

### MCAs Enhance CD86 Expression on Migratory Dendritic Cells in the Draining Cervical Lymph Node After Nasal Vaccination

**Figure 8:**
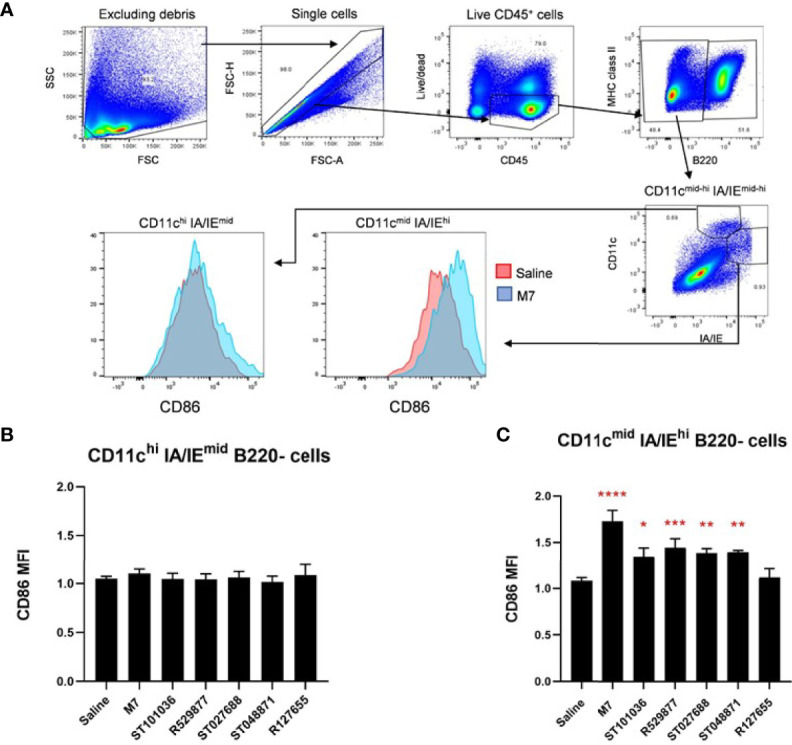
Mast cell activators enhance co-stimulatory molecules on dendritic cells in the draining cervical lymph nodes. Mouse resident and migratory dendritic cells in the draining cervical lymph node were monitored for CD86 expression after exposure to 200 nmoles of the mast cell activating compounds (ST101036, R127655, R529877, ST027688, ST048871), M7 (positive control), or saline (negative control). The gating strategy used to identify the cell populations is presented in **(A)**. Resident DCs are characterized as CD11c^hi^ and MHCII^mid^ **(B)** and Migratory DCs are characterized as CD11c^mid^ and MHCII^hi^ populations **(C)**. Compound-induced CD86 expression is normalized to DCs from mice exposed to saline. One-way ANOVA determined a significant increase compared to saline *p < 0.05, **p < 0.01, ***p < 0.001, ****p < 0.0001. Bars represent the mean + SEM. MFI denotes mean fluorescence intensity.

Although we did not observe an increase in cDCs in the draining CLN after nasal delivery for each small molecule MCA adjuvant, nasally-delivered MCAs adjuvant activity may correlate with enhanced dendritic cell activation, as previously evaluated by our group (54). Therefore, expression of the cell surface costimulatory molecule CD86 was measured on CD11c+ dendritic cells after nasal immunization to determine if the nasal adjuvant activity of small molecule MCA correlated with DC maturation. Mice were nasally administered saline, M7, or the MCA small molecules. Cervical lymph node cells were analyzed for CD86 expression by flow cytometry 24-hours after adjuvant exposure (**Figure 8A**). We did not observe a difference in CD86 expression in CD11c+ resident dendritic cells (CD11c^hi^ IA/IE^mid^ B220^-^) (**Figure 8B**); however, differential CD86 expression was observed in CD11c+ migratory dendritic cells (CD11c^mid^ IA/IE^hi^ B220^-^) (55) (**Figure 8C**). ST101036 (p = 0.02), R529877 (p = 0.001), ST027688 (p = 0.007), ST048871 (p = 0.005), and M7 (p < 0.0001) enhanced CD86 expression on CD11c+ migratory dendritic cells compared to migratory dendritic cells isolated from mice treated with saline. The MCAs that enhance CD86 expression in migratory DC also have nasal adjuvant activity as determined by their ability to induce elevated antigen-specific serum IgG when co-administered with WNV EDIII (**Figure 5**). Therefore, MCA nasal vaccine adjuvant activity correlates with increased CD86 expression in CLN migratory DCs.

## Discussion

Small molecule mast cell activators evaluated for adjuvant activity after nasal immunization were identified by high throughput screening of commercially available compound libraries based on induction of a mast cell degranulation phenotype (23). Although novel small molecule MCAs adjuvant activity is not as effective as that induced by the toll-like receptor 4 (TLR4) ligand MPL, an accepted vaccine adjuvant, for protective immunity in the WNV challenge model, we believe the adjuvant activity demonstrated with the small molecules after nasal delivery is significant, especially when we acknowledge that the compounds are at the hit-to-lead phase of drug discovery. The compounds tested in our *in vivo* immunization studies represent the compounds identified from the original small molecule library and additional reiterative medicinal chemistry as part of the lead optimization phase of drug discovery would likely be required to improve the potency of small molecule mast cell activator vaccine adjuvants. For example, the development of potent novel adjuvants from specific small molecules often requires extensive medical chemistry (56–58) and/or novel formulations (59, 60). Thus, the results presented in this manuscript represent small molecule activators in the hit-to-lead phase of drug discovery and additional medicinal chemistry are needed to determine if the small molecule MCA discussed in this manuscript can be developed to provide safe adjuvant activity *in vivo* and increase vaccine efficacy.

The *in vivo* activity of adjuvants is influenced by their pharmaceutical formulations. For example, liposomes, nanoemulsions, and micro/nanoparticles are used as vaccine delivery systems and can improve the activity of the adjuvants (61–65). Formulation of a tri-adjuvant containing the TLR3 ligand, poly(I:C), the peptide adjuvant IDR-1002, and the inorganic polymer, polyphosphazene, into a liposome improved antigen-specific serum antibody responses and cytokine secreting cells after nasal immunization compared to the tri-adjuvant in the absence of a liposome (66). Similarly, nasal immunization with a formulation of C48/80 in chitosan nanoparticles increases serum lethal toxin neutralizing antibodies compared to immunization with C48/80 in an aqueous formulation (67). In the present study, vaccine formulations that attempted to combine the small molecule MCAs with the antigen in an aqueous vehicle failed due to the hydrophobicity of the MCA resulting in the MCAs precipitating out of solution. Therefore, effective vaccine delivery required procedures that first delivered the MCAs in a formulation of 50% PEG400:water followed by delivery of the antigen in saline. This split delivery method maintained the MCAs in solution and improved compound delivery. However, the maximal adjuvant activity requires co-delivery of the vaccines antigens and adjuvants (68) and it is likely, the less than optimal adjuvant formulation and vaccination method utilized in this study explains the variability within the host immune responses and reduced protection against WNV infection compared to MPL. Therefore, better formulations that co-delivers MCAs and antigens, such as liposomes, nanoemulsions, or micro/nanoparticles may maximize the adjuvant activity of the MCA small molecules after nasal delivery.

Nasal immunization is an effective method to induce mucosal antibody responses (69) but is also a needle-free method of immunization able to induce potent systemic adaptive immune responses (70). Needle-free methods of immunization may be beneficial for individuals with needle-phobia and increase vaccine compliance (71), benefit mass vaccination campaigns (72), and increase vaccine coverage in developing countries (73, 74). Although West Nile virus infects parenterally and vaccine-induced protection against WNV can be mediated by serum antibodies (75), we utilized the WNV vaccine as a proof-of-concept model to evaluate the ability of small molecule MCA to induce antigen-specific systemic immune responses after needle-free nasal immunization. We also measured mucosal WNV-specific antibody responses because we utilized a mucosal route of immunization to evaluate the adjuvant activity of the MCAs; however, we did not detect any mucosal WNV-specific antibody responses in the vaccinated mice, including mice immunized with MPL (data not shown). The antigen dose used in our vaccine formulations may be too low for induction of antigen-specific mucosal antibodies as the antigen dose is an important driver of mucosal immunity (76). However, we selected a low antigen dose in our vaccine studies to increase the sensitivity of evaluating the adjuvant activity of our MCA small molecules. The antigen dose delivered intranasally in this study is lower than the antigen dose used by others in parenteral WNV vaccine studies (77, 78) and we also reported that nasal immunization may require at least three times more antigen as parenteral immunization to induce a similar immune response (21). The vaccine conditions evaluated in this proof-of-concept study may not be optimal for induction of protective immunity against West Nile Virus; however, this study provides a foundation for further evaluation of mast cell activator small molecules as adjuvants for mucosal delivery. However, additional studies are needed to optimize the mast cell activators in nasal vaccines to enhance systemic immunity to protect against systemic pathogens like WNV and induce mucosal immunity that may be beneficial for pathogens that infect mucosal surfaces such as influenza.

Mast cell activators activated dendritic cells *in vitro* and *in vivo*. The small molecule MCA tested in our studies were identified based on their ability to induce mast cell degranulation. However, molecules that activate mast cells may also activate other cell types. We observed enhanced cytokine production, including IL-1, from mouse macrophages, dendritic cells, and lung epithelial cells after exposure to the 15 hit MCAs in the present study, suggesting these compounds can activate cells other than mast cells and IL-1 production from innate cells may contribute to the adjuvant activity of small molecule MCA (17, 39, 70). In a direct comparison between mouse MC/9 mast cells and JAWSII dendritic cells, some compounds induced a stronger cytokine response in JAWSII cells than MC/9 cells, despite being identified based on mast cell degranulation. Dendritic cell activation is a property shared by other known vaccine adjuvants including the TLR ligands MPL and CpG (79). Increased B220+ cell numbers and costimulatory molecule CD86 expression on migratory DCs observed in the draining lymph node after nasal delivery of the small molecule MCAs in this study suggests MCAs activate DCs *in vivo*. Because increased costimulatory molecules on antigen presenting cells is another mechanism adjuvants may use to exert adjuvant activity MCA small molecules may enhance migratory DCs CD86 to provide adjuvant activity. Thus, future studies are required to investigate the role of mast cell activation vs dendritic cell activation in the mechanism of small molecule adjuvant activity after nasal delivery.

Cytotoxicity is a mechanism by which vaccine adjuvants may provide *in vivo* activity (80) and the small molecule MCA tested in this manuscript exhibited a range of cytotoxicity *in vitro*. Although cell death is thought to be an adverse response to a vaccine adjuvant, several vaccine adjuvants induce cell death but maintain appropriate safety for use. Alum is the most widely used vaccine adjuvant and induces cell death, which contributes to its mechanism of action (80). The nanoemulsion vaccine adjuvant W (80)5EC is a potent vaccine adjuvant in mice, rats, and ferrets (81–83), and has been demonstrated safe in humans (84). However, W (80)5EC is cytotoxic in epithelial cells (85), and this cell death is associated with increased cytokine production and dendritic cell activation, which may contribute to its adjuvanticity (86, 87). Vaccine adjuvants that induce localized cytotoxicity may mimic host responses to natural infections. For example, rhinovirus infections induce cytotoxic responses in bronchial epithelial cells and reduce the ability of the epithelial cells to repair themselves (88). Similarly, influenza viral infections commonly induce cell death by a variety of mechanisms including apoptosis, necrosis, and pyroptosis (89, 90), which results in antigen presenting cell activation and recruitment of immune cells with antiviral activities (89). Cytotoxicity may be an initiating event to activate host innate immunity and induce antigen-specific immune responses; thus, compounds with cytotoxic effects should not be excluded from development as vaccine adjuvants.

Small molecule MCAs modified gene expression in the NALT after nasal delivery. RNA sequencing identified several genes enhanced by more than one small molecule MCA, which were also enhanced by M7, suggesting some of the small molecule MCAs may activate host responses using a mechanism similar to that utilized by M7. However, compound R127655 did not share enhanced genes with the other MCAs or M7, suggesting this compound may behave differently than M7 and the other MCAs. Because our RNA sequencing study was performed as a method to evaluate *in vivo* activity of the MCAs, we only measured gene expression at one time point and in one tissue. Although we only selected one time point to evaluate adjuvant-induced gene expression in our studies, we observed enhanced *ccl3* and *manf* expression after exposure to MCAs, which is confirmed by others who have also reported changes in expression *ccl3* and *manf* in mast cell-mediated events (91). Similarly, our observation of enhanced *cxcl10* and *irf7* expression after exposure to MPL confirms the activity of MPL described in the literature (92, 93). Because RNA sequencing studies typically monitor gene expression over several time points and in different tissues to identify biomarkers involved in vaccine and adjuvant activities (94); additional studies are required to better utilize gene expression analysis to identify biomarkers that elucidate MCAs adjuvant activity for safety and efficacy.

The results from this study provide the support that MCAs are a new class of vaccine adjuvants for mucosal immunization. Mast cell activating polymeric compounds (5, 13), peptides (21, 22), and now, small molecules have been shown to enhance vaccine-specific immunity after nasal delivery. The hydrophobic nature of small molecules will require optimal formulations or medicinal chemical modifications to maximize compound activity and advance these compounds through the lead optimization phase of drug discovery. Additional studies are required to identify appropriate adjuvant delivery systems, optimize the adjuvant activity of small molecule MCAs and evaluate safety. However, the use of small molecules as nasal vaccine adjuvants may be a cost-effective method to allow for the rapid production of adjuvant compounds to high purity to be used as a needle-free immunization method.

## Data Availability Statement

Publicly available datasets were analyzed in this study. This data can be found here: https://doi.org/10.7924/r4h133m7w.

## Ethics Statement

The animal study was reviewed and approved by Duke University Institutional Animal Care and Use Committee.

## Author Contributions

BJ-W and HC performed the experiments. BJ-W, HC, CC, HY, and JG performed data analysis. BJ-W, HC, SA, and HS designed the experiments. BJ-W and HS wrote the manuscript. All authors reviewed the manuscript and agree to be accountable for the content of the work. All authors contributed to the article and approved the submitted version.

## Funding

This work was funded by the National Institute of Allergy and Infectious Diseases (NIAID) Adjuvant Discovery Contract #HHSN272201400054C.

## Conflict of Interest

The authors declare that the research was conducted in the absence of any commercial or financial relationships that could be construed as a potential conflict of interest.

## Publisher’s Note

All claims expressed in this article are solely those of the authors and do not necessarily represent those of their affiliated organizations, or those of the publisher, the editors and the reviewers. Any product that may be evaluated in this article, or claim that may be made by its manufacturer, is not guaranteed or endorsed by the publisher.
