# Supplementary material for: Nasal Immunization With Small Molecule Mast Cell Activators Enhance Immunity to Co-Administered Subunit Immunogens
Source: Front Immunol. 2021 Sep 10;12:730346. doi: 10.3389/fimmu.2021.730346 (PMC8461742; doi:10.3389/fimmu.2021.730346)
Supplement: Supplementary file 1 [file Table_1.docx]

| **Table S1. Compound-induced cytokine production from MC/9 mast cells** | | | | | | | | | | | | | | | | | | | |
| --- | --- | --- | --- | --- | --- | --- | --- | --- | --- | --- | --- | --- | --- | --- | --- | --- | --- | --- | --- |
|  | **Media** | **MPL** | **C48/80** | **M7-NH2** | **L147192** | **L201863** | **R127655** | **R529877** | **R606278** | **ST026567** | **ST027688** | **ST029248** | **ST029279** | **ST045940** | **ST048871** | **ST081379** | **ST086136** | **ST099914** | **ST101036** |
| **Eotaxin** | 0.0 | 0.0 | 0.0 | 0.0 | 0.0 | 0.0 | 0.0 | 0.0 | 0.0 | 0.0 | 0.0 | 0.0 | 0.0 | 0.0 | 0.0 | 0.0 | 0.0 | 0.0 | 0.0 |
| **GCSF** | 0.0 | 0.0 | 0.0 | 0.0 | 0.0 | 0.0 | 0.0 | 0.0 | 0.0 | 0.0 | 0.0 | 0.0 | 0.0 | 0.0 | 0.0 | 0.0 | 0.0 | 0.0 | 0.0 |
| **GMCSF** | 0.0 | -4.1 | -4.1 | -4.1 | -4.1 | -4.1 | 0.5 | 1.4 | -4.1 | -4.1 | **3.5** | -4.1 | **5.8** | -4.1 | -4.1 | -4.1 | **3.1** | -4.1 | 0.7 |
| **IFNg** | 0.0 | 0.0 | 0.0 | 0.0 | 0.0 | 0.0 | 0.0 | 0.0 | 0.0 | 0.0 | 0.0 | 0.0 | 0.0 | 0.0 | 0.0 | 0.0 | 1.0 | 0.0 | 0.0 |
| **IL10** | 0.0 | 0.0 | 0.0 | 0.0 | 0.0 | 0.0 | 0.7 | 0.0 | 0.0 | 0.0 | **2.7** | 0.0 | 0.9 | 0.0 | **2.8** | 0.0 | **2.9** | 0.0 | 0.0 |
| **IL12p40** | 0.0 | 0.8 | 0.0 | 0.0 | 0.0 | 0.0 | 0.0 | 0.0 | 0.0 | 0.0 | 0.0 | 0.0 | 0.0 | 0.0 | 0.0 | 0.0 | 0.0 | 0.0 | 0.0 |
| **IL12p70** | 0.0 | 0.0 | 0.0 | 0.0 | 0.0 | 0.0 | 0.0 | 0.0 | 0.0 | 0.0 | 0.0 | 0.0 | 0.0 | 0.0 | 0.0 | 0.0 | 0.0 | 0.0 | 0.0 |
| **IL13** | 0.0 | 0.0 | 0.0 | 0.0 | 0.0 | 0.0 | 0.0 | 0.0 | 0.0 | 0.0 | 0.0 | 0.0 | 0.0 | 0.0 | 0.0 | 0.0 | 0.0 | 0.0 | 0.0 |
| **IL15** | 0.0 | 1.6 | -0.4 | 1.8 | -1.5 | -1.5 | -1.5 | -1.5 | -1.5 | **3.2** | **4.4** | -1.5 | -0.4 | -1.5 | 0.6 | -1.5 | 0.7 | -0.7 | -1.5 |
| **IL17** | 0.0 | 0.3 | **8.1** | 0.2 | -8.2 | -7.5 | -4.3 | **3.6** | **33.6** | **19.3** | -0.4 | **4.1** | **34.9** | -3.0 | **2.0** | **32.3** | -7.1 | -5.5 | **32.2** |
| **IL1a** | 0.0 | **9.9** | **87.0** | **6.1** | 0.0 | 0.0 | 0.0 | 0.0 | 0.0 | **7.7** | 0.0 | 0.0 | 0.0 | 0.0 | **11.6** | 0.0 | **20.7** | 0.0 | 0.0 |
| **IL1b** | 0.0 | 0.9 | -0.9 | -0.9 | -5.1 | -5.1 | **3.7** | **4.9** | -0.9 | -0.9 | -0.2 | -5.1 | -5.1 | -0.9 | -0.7 | -5.1 | **8.5** | -0.9 | -5.1 |
| **IL2** | 0.0 | 0.9 | **8.1** | 0.0 | 0.0 | 0.0 | 0.0 | 0.0 | 0.0 | 0.0 | 0.0 | 0.0 | 0.0 | 0.0 | 0.0 | 0.0 | **2.2** | 0.0 | 0.0 |
| **IL3** | 0.0 | 0.0 | 0.0 | 0.0 | 0.0 | 0.0 | 0.0 | 0.0 | 0.0 | 0.9 | 0.0 | 0.0 | 0.0 | 0.0 | 0.0 | 0.0 | 0.0 | 0.0 | 0.0 |
| **IL4** | 0.0 | 0.0 | 0.0 | 0.0 | 0.0 | 0.0 | 0.0 | 0.0 | 0.0 | 0.0 | 0.0 | 0.0 | 0.0 | 0.0 | 0.0 | 0.0 | 0.0 | 0.0 | 0.0 |
| **IL5** | 0.0 | -0.4 | -5.7 | -3.1 | -4.8 | -5.7 | -4.7 | -5.7 | -5.7 | -5.7 | -1.4 | -5.7 | -5.0 | -5.7 | -5.7 | -5.7 | -2.5 | -5.7 | -5.7 |
| **IL6** | 0.0 | **506.4** | -567.1 | -541.6 | -483.4 | -39.3 | -319.9 | -451.6 | -409.9 | **110.0** | **5322.7** | -568.8 | -400.6 | -415.2 | **1480.2** | -568.6 | -509.4 | -405.8 | -562.0 |
| **IL7** | 0.0 | 0.0 | 0.0 | 0.0 | 0.0 | 0.0 | 0.0 | 0.0 | 0.0 | 0.8 | 0.0 | 0.0 | 0.0 | 0.0 | 0.0 | 0.0 | 0.0 | 0.0 | 0.0 |
| **IL9** | 0.0 | -26.8 | **307.8** | **27.8** | **305.2** | **109.9** | -19.1 | -2.2 | **161.2** | **176.5** | -21.1 | -6.0 | **187.3** | -7.5 | -27.4 | **197.5** | **105.9** | **86.9** | **133.0** |
| **IP10** | 0.0 | -7.0 | **17.3** | -4.7 | -19.0 | -36.2 | -36.3 | **29.3** | -10.4 | -34.1 | **21.0** | **20.8** | **28.2** | -35.3 | **36.3** | -11.3 | -40.2 | -4.8 | -38.4 |
| **KC** | 0.0 | 1.6 | 0.0 | 0.0 | **2.4** | 1.8 | **2.0** | 0.0 | 0.8 | 0.0 | 0.0 | 0.0 | 0.8 | 0.0 | 0.0 | 1.8 | 1.9 | **2.3** | **2.0** |
| **LIF** | 0.0 | -35.8 | **192.8** | **369.1** | -545.1 | -446.2 | -560.8 | -222.6 | -515.3 | **373.8** | -158.3 | -246.2 | -408.9 | -529.7 | -135.3 | -591.0 | -547.5 | -519.4 | -488.5 |
| **LIX** | 0.0 | 0.0 | **14.1** | 0.0 | 0.0 | 0.0 | 0.0 | 0.0 | 0.0 | 0.0 | **3.8** | 0.0 | **3.4** | 0.0 | 0.0 | 0.0 | 0.0 | 0.0 | 0.0 |
| **MCSF** | 0.0 | -27.6 | -80.9 | **121.4** | -90.0 | -79.4 | -89.1 | -37.5 | -73.8 | -54.7 | -37.5 | -37.5 | -54.1 | -80.8 | -21.7 | -91.4 | -86.3 | -81.6 | -74.4 |
| **MCP1** | 0.0 | -219.8 | -5035.5 | -3332.2 | -2651.6 | -3806.0 | -2017.0 | -4442.5 | -4773.0 | -4684.1 | -2533.8 | -4696.8 | -4658.2 | -4669.9 | -3116.9 | -4674.0 | -3462.5 | -3116.3 | -4686.1 |
| **MIG** | 0.0 | **2.1** | **14.2** | **12.2** | **23.9** | **20.7** | **19.1** | **3.8** | **22.2** | **21.2** | **3.2** | **4.2** | **26.0** | **19.7** | **7.7** | **19.6** | **20.7** | **19.7** | **18.8** |
| **MIP1a** | 0.0 | **59.6** | -284.6 | -295.5 | -266.2 | -142.7 | -228.8 | -286.8 | -270.2 | **449.2** | **1259.6** | -307.0 | -230.7 | -222.1 | **222.4** | -325.9 | -281.1 | -207.8 | -325.2 |
| **MIP1b** | 0.0 | **55.6** | -226.1 | -228.0 | -212.9 | -107.5 | -194.8 | -217.6 | -204.9 | **208.1** | **1108.0** | -224.7 | -190.7 | -181.9 | **14.8** | -233.2 | -216.7 | -166.4 | -234.2 |
| **MIP2** | 0.0 | 0.9 | **35.4** | -0.5 | -6.1 | 0.5 | **11.1** | -6.1 | -6.1 | -6.1 | 0.0 | **23.6** | -6.1 | -6.1 | -6.1 | 0.5 | **20.7** | -1.0 | -1.0 |
| **RANTES** | 0.0 | -0.6 | -3.3 | -1.0 | -2.0 | -2.4 | -2.2 | -0.8 | 0.6 | 1.1 | 0.7 | -0.8 | 1.3 | -1.7 | 0.6 | 0.7 | -2.7 | -1.4 | 0.4 |
| **TNFa** | 0.0 | **11.1** | -24.0 | -19.8 | -17.4 | -7.1 | -10.1 | -10.9 | -7.2 | **18.8** | **171.4** | -23.0 | -4.2 | -13.0 | **32.6** | -18.6 | -16.9 | -17.1 | -19.7 |
| **VEGF** | 0.0 | 1.2 | -8.1 | -7.7 | -11.4 | -12.2 | -8.6 | -13.4 | -19.4 | -20.2 | **15.4** | -15.2 | -17.1 | -21.1 | **20.4** | -20.3 | -6.0 | -16.0 | -21.1 |
| Mouse MC/9 mast cells were stimulated with the 15 hit MCAs or with MPL, C48/80, or M7 as positive controls or in media without stimuli as a negative control for 24-hours. Cell supernatants were collected and measured for secreted cytokine production using a cytokine/chemokine multiplex assay. Cytokine/chemokine values for cells in media were subtracted from cells stimulated with the 15 MCAs or controls. Red values indicate a positive pg/ml value after pg/ml values in media was subtracted. | | | | | | | | | | | | | | | | | | | |

| **Table S2. Compound-induced cytokine production from LA-4 lung epithelial cells** | | | | | | | | | | | | | | | | | | | |
| --- | --- | --- | --- | --- | --- | --- | --- | --- | --- | --- | --- | --- | --- | --- | --- | --- | --- | --- | --- |
|  | **Media** | **MPL** | **C48/80** | **M7-NH2** | **L147192** | **L201863** | **R127655** | **R529877** | **R606278** | **ST026567** | **ST027688** | **ST029248** | **ST029279** | **ST045940** | **ST048871** | **ST081379** | **ST086136** | **ST099914** | **ST101036** |
| **Eotaxin** | 0.0 | 0.0 | 0.0 | 0.0 | 0.0 | 0.0 | 0.0 | 0.0 | 0.0 | 0.0 | 0.0 | 0.0 | 0.0 | 0.0 | 0.0 | 0.0 | 0.0 | 0.0 | 0.0 |
| **GCSF** | 0.0 | **7.7** | 0.0 | 0.0 | 0.0 | 0.0 | 0.0 | 0.0 | 0.0 | 0.0 | 0.0 | 0.0 | 0.0 | 0.0 | 0.0 | 0.0 | 0.0 | 0.0 | 0.0 |
| **GMCSF** | 0.0 | **6.2** | 0.0 | -0.5 | **5.9** | **1.0** | **6.9** | 0.0 | -1.8 | -5.9 | **10.6** | -1.8 | **24.1** | -5.9 | **5.9** | **19.7** | **3.6** | -5.9 | -5.9 |
| **IFNg** | 0.0 | 0.0 | 0.0 | 0.0 | 0.0 | 0.0 | 0.0 | 0.0 | 0.0 | 0.0 | 0.0 | 0.0 | 0.0 | 0.0 | 0.0 | **0.7** | 0.0 | 0.0 | 0.0 |
| **IL10** | 0.0 | **0.6** | **6.0** | **0.7** | **1.4** | **0.6** | -0.1 | -0.1 | **0.4** | -0.1 | -0.7 | -0.7 | **1.0** | -0.1 | -0.7 | **1.7** | **0.3** | **0.4** | **0.2** |
| **IL12p40** | 0.0 | 0.0 | 0.0 | 0.0 | 0.0 | 0.0 | 0.0 | 0.0 | 0.0 | 0.0 | 0.0 | 0.0 | 0.0 | 0.0 | 0.0 | 0.0 | 0.0 | 0.0 | 0.0 |
| **IL12p70** | 0.0 | 0.0 | 0.0 | 0.0 | 0.0 | 0.0 | 0.0 | 0.0 | 0.0 | 0.0 | 0.0 | 0.0 | 0.0 | 0.0 | 0.0 | 0.0 | 0.0 | 0.0 | 0.0 |
| **IL13** | 0.0 | 0.0 | 0.0 | 0.0 | 0.0 | 0.0 | 0.0 | 0.0 | 0.0 | 0.0 | 0.0 | 0.0 | 0.0 | 0.0 | 0.0 | 0.0 | 0.0 | 0.0 | 0.0 |
| **IL15** | 0.0 | -0.1 | **2.7** | **3.0** | **5.6** | **1.7** | -0.9 | **3.3** | **0.4** | **1.4** | -0.1 | -0.4 | **0.3** | -2.7 | **0.3** | **1.8** | **2.6** | **4.0** | -1.6 |
| **IL17** | 0.0 | 0.0 | 0.0 | 0.0 | 0.0 | 0.0 | 0.0 | 0.0 | 0.0 | 0.0 | 0.0 | 0.0 | 0.0 | 0.0 | 0.0 | 0.0 | 0.0 | 0.0 | 0.0 |
| **IL1a** | 0.0 | **25.7** | **141.1** | **4.6** | **26.1** | **3.1** | **6.4** | **14.5** | **3.1** | **3.1** | **16.0** | **4.2** | **3.6** | **9.7** | 0.0 | **4.2** | **4.2** | 0.0 | **67.4** |
| **IL1b** | 0.0 | -7.8 | **7.1** | -1.5 | -10.7 | -7.6 | -3.0 | -7.6 | -0.6 | -3.3 | -3.5 | -7.8 | -10.7 | -5.1 | -10.7 | -10.7 | -6.8 | -3.5 | -7.6 |
| **IL2** | 0.0 | 0.0 | **1.0** | 0.0 | 0.0 | 0.0 | 0.0 | 0.0 | 0.0 | 0.0 | 0.0 | 0.0 | **0.9** | 0.0 | 0.0 | 0.0 | 0.0 | 0.0 | **5.7** |
| **IL3** | 0.0 | 0.0 | 0.0 | 0.0 | 0.0 | 0.0 | 0.0 | 0.0 | 0.0 | 0.0 | 0.0 | 0.0 | 0.0 | 0.0 | 0.0 | 0.0 | 0.0 | 0.0 | 0.0 |
| **IL4** | 0.0 | 0.0 | 0.0 | 0.0 | 0.0 | 0.0 | 0.0 | 0.0 | 0.0 | 0.0 | 0.0 | 0.0 | 0.0 | 0.0 | 0.0 | 0.0 | 0.0 | 0.0 | 0.0 |
| **IL5** | 0.0 | **1.8** | -9.4 | -9.3 | **2.5** | -12.5 | **2.1** | -3.3 | -11.0 | -11.3 | **2.7** | -12.5 | -2.3 | -11.3 | -4.1 | -8.4 | **0.6** | **0.3** | -11.7 |
| **IL6** | 0.0 | **23.2** | -35.7 | -18.6 | -11.6 | -37.7 | **56.0** | **14.2** | -37.7 | -35.7 | -19.4 | -37.7 | -25.9 | -37.7 | **92.4** | -35.8 | -8.3 | -9.4 | -37.7 |
| **IL7** | 0.0 | **0.6** | -1.1 | -1.1 | **1.0** | **0.8** | -1.1 | -1.1 | -1.1 | -1.1 | -0.2 | -1.1 | -1.1 | -1.1 | -1.1 | -1.1 | -1.1 | **0.6** | -0.3 |
| **IL9** | 0.0 | 0.0 | 0.0 | 0.0 | 0.0 | 0.0 | 0.0 | 0.0 | 0.0 | 0.0 | 0.0 | 0.0 | 0.0 | 0.0 | 0.0 | 0.0 | 0.0 | 0.0 | 0.0 |
| **IP10** | 0.0 | **136.5** | -105.6 | -209.9 | -21.1 | -290.9 | -122.3 | -195.8 | -291.0 | -289.9 | -77.1 | -252.1 | -202.1 | -292.4 | -188.8 | -204.6 | -227.8 | -94.3 | -214.5 |
| **KC** | 0.0 | **71485.3** | -5746.5 | -3429.1 | **1499.9** | -5589.2 | **890.8** | -1331.5 | -5821.0 | -5648.1 | -42.9 | -5621.4 | **43.3** | -5729.6 | -2311.6 | -4813.0 | -1304.5 | **47224.5** | -5280.1 |
| **LIF** | 0.0 | **105.6** | -31.5 | -28.1 | -5.8 | -29.3 | **1.0** | **64.4** | -14.3 | -26.5 | -10.3 | -31.5 | **51.6** | -31.5 | **15.8** | **37.8** | **10.1** | -20.7 | -31.5 |
| **LIX** | 0.0 | **362.7** | -436.5 | -429.9 | -227.5 | -448.2 | -266.9 | -413.6 | -448.2 | -432.2 | -216.0 | -448.2 | -391.9 | -434.0 | -448.2 | -448.2 | -320.3 | -60.4 | -448.2 |
| **MCSF** | 0.0 | **3.4** | **2.0** | -2.3 | **0.4** | -5.1 | **2.2** | **193.0** | **26.1** | **573.3** | -1.2 | -5.7 | **56.5** | -4.4 | **2.9** | **697.3** | **70.9** | -2.2 | **1.3** |
| **MCP1** | 0.0 | **95114.1** | -4867.2 | -3819.8 | -1134.2 | -4786.4 | -658.3 | -2609.6 | -4880.6 | -4845.2 | **262.5** | -4774.4 | -2195.1 | -4802.2 | -3299.6 | -3998.2 | -1768.9 | -951.2 | -4549.7 |
| **MIG** | 0.0 | 0.0 | **55.4** | 0.0 | **5.5** | 0.0 | 0.0 | 0.0 | 0.0 | 0.0 | 0.0 | 0.0 | 0.0 | 0.0 | 0.0 | 0.0 | 0.0 | **9.0** | 0.0 |
| **MIP1a** | 0.0 | **5.3** | **26.8** | **4.1** | **2.7** | **1.4** | **1.9** | -5.8 | **1.9** | -3.1 | -3.5 | -0.5 | -4.9 | -3.4 | -0.1 | -5.1 | **3.1** | **1.5** | **9.7** |
| **MIP1b** | 0.0 | -0.9 | **1.1** | -0.9 | **0.9** | -0.9 | **0.2** | -0.9 | **1.0** | **1.6** | -0.9 | -0.9 | **0.4** | **0.1** | -0.9 | **1.2** | **0.7** | **1.5** | -0.9 |
| **MIP2** | 0.0 | 0.0 | 0.0 | 0.0 | 0.0 | 0.0 | 0.0 | 0.0 | 0.0 | 0.0 | 0.0 | 0.0 | 0.0 | 0.0 | 0.0 | 0.0 | 0.0 | 0.0 | 0.0 |
| **RANTES** | 0.0 | **122.7** | -145.8 | -125.9 | -42.0 | -145.8 | -4.4 | -111.2 | -145.8 | -145.8 | -46.4 | -144.3 | -129.3 | -145.8 | -114.8 | -141.1 | -59.4 | -18.0 | -141.9 |
| **TNFa** | 0.0 | 0.0 | 0.0 | 0.0 | 0.0 | 0.0 | 0.0 | 0.0 | 0.0 | 0.0 | 0.0 | 0.0 | 0.0 | 0.0 | 0.0 | 0.0 | 0.0 | 0.0 | 0.0 |
| **VEGF** | 0.0 | **148.7** | -540.6 | -323.6 | **80.5** | -215.4 | **186.2** | -70.4 | -547.7 | -88.7 | **110.8** | -543.0 | -137.5 | -352.9 | **264.8** | -455.1 | **369.2** | -157.8 | -524.8 |
| Mouse LA-4 lung epithelial cells were stimulated with the 15 hit MCAs or with MPL, C48/80, or M7 as positive controls or in media without stimuli as a negative control for 24-hours. Cell supernatants were collected and measured for secreted cytokine production using a cytokine/chemokine multiplex assay. Cytokine/chemokine values for cells in media were subtracted from cells stimulated with the 15 MCAs or controls. Red values indicate a positive pg/ml value after pg/ml values in media was subtracted. | | | | | | | | | | | | | | | | | | | |

| **Table S3. Compound-induced cytokine production from J774A.1 monocyte/macrophages** | | | | | | | | | | | | | | | | | | | |
| --- | --- | --- | --- | --- | --- | --- | --- | --- | --- | --- | --- | --- | --- | --- | --- | --- | --- | --- | --- |
|  | **Media** | **MPL** | **C48/80** | **M7-NH2** | **L147192** | **L201863** | **R127655** | **R529877** | **R606278** | **ST026567** | **ST027688** | **ST029248** | **ST029279** | **ST045940** | **ST048871** | **ST081379** | **ST086136** | **ST099914** | **ST101036** |
| **Eotaxin** | 0.0 | **2.4** | 0.4 | -0.3 | -0.3 | -1.7 | -0.3 | -0.6 | -4.1 | 0.4 | 0.4 | -2.5 | -4.1 | -2.5 | -0.4 | -3.4 | -0.3 | -0.3 | -4.1 |
| **GCSF** | 0.0 | **99998.4** | -1.5 | -1.5 | **2.5** | -1.5 | -0.4 | -1.5 | -1.5 | -0.5 | **6.0** | -1.5 | -1.5 | -1.5 | **31.6** | -1.5 | -1.5 | -0.5 | -1.5 |
| **GMCSF** | 0.0 | **21.7** | **2.2** | -2.3 | 0.0 | -3.9 | 1.3 | -1.4 | -2.3 | -0.1 | 2.0 | -1.1 | -3.0 | -2.2 | -0.5 | -3.2 | -1.1 | -3.9 | -3.0 |
| **IFNg** | 0.0 | 0.0 | 0.0 | 0.0 | 0.0 | 0.0 | 0.0 | 0.0 | 0.0 | 0.0 | 0.0 | 0.0 | 0.0 | 0.0 | 0.0 | 0.0 | 0.0 | 0.0 | 0.0 |
| **IL10** | 0.0 | **43.1** | 0.0 | -1.5 | -0.7 | -1.5 | -1.5 | -1.5 | -1.5 | -1.5 | 0.9 | -1.5 | -0.7 | -1.5 | -0.8 | -1.5 | -1.5 | -1.5 | -1.5 |
| **IL12p40** | 0.0 | 0.0 | 0.0 | 0.0 | 0.0 | 0.0 | 0.0 | 0.0 | 0.0 | 0.0 | 0.0 | 0.0 | 0.0 | 0.0 | 0.0 | 0.0 | 0.0 | 0.0 | 0.0 |
| **IL12p70** | 0.0 | 0.0 | 0.0 | 0.0 | 0.0 | 0.0 | 0.0 | 0.0 | 0.0 | 0.0 | 0.0 | 0.0 | 0.0 | 0.0 | 0.0 | 0.0 | 0.0 | 0.0 | 0.0 |
| **IL13** | 0.0 | 0.0 | 0.0 | 0.0 | 0.0 | 0.0 | 0.0 | 0.0 | 0.0 | 0.0 | 0.0 | 0.0 | 0.0 | 0.0 | 0.0 | 0.0 | 0.0 | 0.0 | 0.0 |
| **IL15** | 0.0 | 1.7 | -0.4 | -2.8 | -1.0 | -3.6 | -0.9 | -2.9 | -3.6 | -0.2 | -0.2 | -3.6 | -3.6 | -3.6 | -1.6 | -3.6 | -3.6 | -2.1 | -3.6 |
| **IL17** | 0.0 | **2.6** | -3.8 | -3.8 | -0.9 | -3.8 | -1.1 | -3.8 | -3.8 | -0.1 | -0.7 | -3.8 | -3.8 | -3.8 | -3.8 | -3.8 | -2.1 | -3.0 | -3.8 |
| **IL1a** | 0.0 | **31.8** | **26.6** | **3.1** | 0.8 | 0.0 | 0.0 | **4.0** | -0.8 | 0.0 | -0.8 | 1.0 | **4.6** | -0.8 | 1.5 | **4.4** | 0.0 | -0.8 | **4.9** |
| **IL1b** | 0.0 | **27.6** | **3.4** | 0.5 | -1.5 | -1.4 | -1.7 | 1.0 | 1.2 | -0.6 | 0.6 | -1.7 | -0.7 | 0.9 | 1.1 | -1.0 | 0.6 | -1.0 | -0.9 |
| **IL2** | 0.0 | 0.0 | 0.0 | 0.0 | 0.0 | 0.0 | 0.0 | 0.0 | 0.0 | 0.0 | 0.0 | 0.0 | 0.0 | 0.0 | 0.0 | 0.0 | 0.0 | 0.0 | 0.0 |
| **IL3** | 0.0 | 0.0 | 0.0 | 0.0 | 0.0 | 0.0 | 0.0 | 0.0 | 0.0 | 0.0 | 0.0 | 0.0 | 0.0 | 0.0 | 0.0 | 0.0 | 0.0 | 0.0 | 0.0 |
| **IL4** | 0.0 | 0.0 | 0.0 | 0.0 | 0.0 | 0.0 | 0.0 | 0.0 | 0.0 | 0.0 | 0.0 | 0.0 | 0.0 | 0.0 | 0.0 | 0.0 | 0.0 | 0.0 | 0.0 |
| **IL5** | 0.0 | 0.9 | 0.0 | 0.0 | 0.0 | 0.0 | 0.0 | 0.0 | 0.0 | 0.0 | 0.0 | 0.0 | 0.0 | 0.0 | 0.0 | 0.0 | 0.0 | 0.0 | 0.0 |
| **IL6** | 0.0 | **99998.8** | **7.5** | **3.2** | **11.6** | -1.1 | 0.0 | **3.5** | -1.1 | -1.1 | **8.5** | -1.1 | **4.7** | 1.0 | **169.5** | -1.1 | -1.1 | -1.1 | -1.1 |
| **IL7** | 0.0 | 0.0 | 0.0 | 0.0 | 0.0 | 0.0 | 0.0 | 0.0 | 0.0 | 0.0 | 0.0 | 0.0 | 0.0 | 0.0 | 0.0 | 0.0 | 0.0 | 0.0 | 0.0 |
| **IL9** | 0.0 | **49.1** | **121.1** | 0.0 | 0.0 | 0.0 | 0.0 | 0.0 | 0.0 | 0.0 | 0.0 | 0.0 | 0.0 | 0.0 | 0.0 | 0.0 | 0.0 | 0.0 | 0.0 |
| **IP10** | 0.0 | **1259.6** | -3.8 | **5.1** | -7.7 | -9.9 | -5.3 | -9.7 | -9.5 | **16.7** | **22.1** | -11.1 | -14.7 | -9.2 | **2.5** | -11.2 | -9.9 | -9.4 | -10.1 |
| **KC** | 0.0 | **10.6** | 0.0 | 0.0 | 0.0 | 0.0 | 0.0 | 0.0 | 0.0 | 0.0 | 0.0 | 0.0 | 0.0 | 0.0 | 0.0 | 0.0 | 0.0 | 0.0 | 0.0 |
| **LIF** | 0.0 | **9.2** | 0.0 | 0.0 | 0.0 | 0.0 | 0.0 | 0.0 | 0.0 | 0.0 | 0.0 | 0.0 | 0.0 | 0.0 | 0.0 | 0.0 | 0.0 | 0.0 | 0.0 |
| **LIX** | 0.0 | **1590.8** | -81.0 | -128.7 | -73.1 | -154.4 | **60.4** | -134.8 | **190.7** | -139.0 | -24.0 | -154.4 | -119.9 | **355.7** | **585.1** | -154.4 | **171.9** | -154.4 | -154.4 |
| **MCSF** | 0.0 | **4.7** | **4.8** | 0.0 | 0.0 | 0.0 | 0.0 | 0.0 | 0.0 | 0.0 | 0.0 | 0.0 | 0.0 | 0.0 | 0.9 | 0.0 | 0.0 | 0.0 | 0.0 |
| **MCP1** | 0.0 | **98511.4** | -1417.0 | -568.4 | -533.9 | -1321.6 | -296.7 | -1223.5 | -1335.7 | -590.2 | **145.4** | -1274.9 | -1427.6 | -1285.8 | -1140.7 | -1322.5 | -1141.0 | -1191.6 | -1261.0 |
| **MIG** | 0.0 | **8.5** | 0.0 | 0.9 | -0.8 | 0.1 | -0.8 | -0.8 | -0.8 | -0.8 | -0.8 | -0.8 | -0.8 | -0.8 | 0.1 | -0.8 | -0.8 | -0.8 | -0.8 |
| **MIP2** | 0.0 | **75079.1** | -1858.2 | -1285.8 | -910.7 | -2251.5 | **1012.7** | -1703.1 | **3912.1** | -1687.2 | **206.1** | -2166.5 | -623.1 | **5341.2** | **97624.4** | -1679.8 | **2771.9** | -1934.9 | -2008.0 |
| **RANTES** | 0.0 | **99999.9** | 0.0 | 0.0 | 0.0 | 0.0 | 0.0 | 0.0 | 0.0 | 0.0 | 0.0 | 0.0 | 0.0 | 0.0 | 0.0 | 0.0 | 0.0 | 0.0 | 0.0 |
| **TNFa** | 0.0 | **99975.0** | -25.0 | -12.4 | -9.1 | -15.2 | **5.6** | -7.5 | **33.0** | -5.1 | **42.7** | -18.7 | -5.8 | **34.1** | **29.2** | -13.2 | -7.6 | -19.2 | -15.4 |
| **VEGF** | 0.0 | 0.0 | 0.8 | 0.0 | 0.0 | 0.0 | 0.0 | 0.0 | 0.0 | 0.0 | 0.0 | 0.0 | 0.0 | 0.0 | 0.0 | 0.0 | 0.0 | 0.0 | 0.0 |
| Mouse J774A.1 monocytes/macrophages were stimulated with the 15 hit MCAs or with MPL, C48/80, or M7 as positive controls or in media without stimuli as a negative control for 24-hours. Cell supernatants were collected and measured for secreted cytokine production using a cytokine/chemokine multiplex assay. Cytokine/chemokine values for cells in media were subtracted from cells stimulated with the 15 MCAs or controls. Red values indicate a positive pg/ml value after pg/ml values in media was subtracted. | | | | | | | | | | | | | | | | | | | |

| **Table S4. Compound-induced cytokine production from JAWSII monocyte/dendritic cells** | | | | | | | | | | | | | | | | | | | |
| --- | --- | --- | --- | --- | --- | --- | --- | --- | --- | --- | --- | --- | --- | --- | --- | --- | --- | --- | --- |
|  | **Media** | **MPL** | **C48/80** | **M7-NH2** | **L147192** | **L201863** | **R127655** | **R529877** | **R606278** | **ST026567** | **ST027688** | **ST029248** | **ST029279** | **ST045940** | **ST048871** | **ST081379** | **ST086136** | **ST099914** | **ST101036** |
| **Eotaxin** | 0.0 | 0.0 | 0.0 | 0.0 | 0.0 | 0.0 | 0.0 | 0.0 | 0.0 | 0.0 | 0.0 | 0.0 | 0.0 | 0.0 | 0.0 | 0.0 | 0.0 | 0.0 | 0.0 |
| **GCSF** | 0.0 | **99961.9** | -31.9 | **553.8** | **70.0** | -16.1 | **66.0** | **2264.0** | **522.7** | **3.2** | -10.5 | -34.8 | **1168.5** | **19.2** | **304.4** | -30.3 | **94.3** | **100.6** | -22.9 |
| **GMCSF** | 0.0 | **111.9** | **7144.1** | **3779.4** | -196.7 | **2090.9** | -187.3 | **964.4** | **1726.1** | **3640.5** | **16.2** | **6263.6** | **2500.6** | **449.6** | **3099.8** | **4843.9** | -20.9 | **645.2** | **7628.4** |
| **IFNg** | 0.0 | **8.9** | 0.0 | 0.0 | 0.0 | 0.0 | 0.0 | **1.8** | 0.0 | 0.0 | 0.0 | 0.0 | **3.0** | 0.0 | **2.0** | 0.0 | 0.0 | 0.0 | **0.9** |
| **IL10** | 0.0 | **12.3** | 0.0 | 0.0 | 0.0 | 0.0 | 0.0 | **4.6** | **2.0** | 0.0 | 0.0 | 0.0 | **5.3** | 0.0 | 0.0 | 0.0 | 0.0 | **3.9** | 0.0 |
| **IL12p40** | 0.0 | **0.8** | 0.0 | 0.0 | 0.0 | 0.0 | 0.0 | 0.0 | 0.0 | 0.0 | 0.0 | 0.0 | 0.0 | 0.0 | 0.0 | 0.0 | 0.0 | 0.0 | 0.0 |
| **IL12p70** | 0.0 | **14.3** | 0.0 | 0.0 | 0.0 | 0.0 | 0.0 | 0.0 | 0.0 | 0.0 | 0.0 | 0.0 | 0.0 | 0.0 | 0.0 | 0.0 | 0.0 | 0.0 | 0.0 |
| **IL13** | 0.0 | **18.9** | 0.0 | 0.0 | 0.0 | 0.0 | 0.0 | 0.0 | 0.0 | 0.0 | 0.0 | 0.0 | 0.0 | 0.0 | 0.0 | 0.0 | 0.0 | 0.0 | 0.0 |
| **IL15** | 0.0 | **24.4** | 0.0 | 0.0 | 0.0 | 0.0 | 0.0 | **12.9** | **1.0** | 0.0 | 0.0 | 0.0 | **4.4** | 0.0 | **2.6** | 0.0 | 0.0 | 0.0 | **1.0** |
| **IL17** | 0.0 | **5.6** | 0.0 | 0.0 | 0.0 | 0.0 | 0.0 | **2.5** | 0.0 | 0.0 | 0.0 | 0.0 | 0.0 | 0.0 | 0.0 | 0.0 | 0.0 | 0.0 | 0.0 |
| **IL1a** | 0.0 | **2149.2** | **60.1** | **450.8** | **108.1** | **99.9** | **82.0** | **819.9** | **570.0** | **153.5** | **83.4** | **381.8** | **878.0** | **108.3** | **3477.4** | **571.8** | **226.4** | **161.5** | **1483.1** |
| **IL1b** | 0.0 | **656.3** | -12.7 | **6.1** | -10.4 | -3.4 | -5.5 | **29.0** | **17.6** | -6.0 | -4.0 | -1.2 | **30.7** | **4.5** | **1022.4** | **154.4** | **1.2** | **7.5** | **18.8** |
| **IL2** | 0.0 | **4.2** | 0.0 | 0.0 | 0.0 | 0.0 | 0.0 | 0.0 | 0.0 | 0.0 | 0.0 | 0.0 | 0.0 | 0.0 | 0.0 | 0.0 | 0.0 | 0.0 | **0.9** |
| **IL3** | 0.0 | 0.0 | 0.0 | 0.0 | 0.0 | 0.0 | 0.0 | 0.0 | 0.0 | 0.0 | 0.0 | 0.0 | 0.0 | 0.0 | 0.0 | 0.0 | 0.0 | 0.0 | 0.0 |
| **IL4** | 0.0 | 0.0 | 0.0 | 0.0 | 0.0 | 0.0 | 0.0 | 0.0 | 0.0 | 0.0 | 0.0 | 0.0 | 0.0 | 0.0 | 0.0 | 0.0 | 0.0 | 0.0 | 0.0 |
| **IL5** | 0.0 | **5.4** | 0.0 | 0.0 | 0.0 | 0.0 | 0.0 | 0.0 | 0.0 | 0.0 | 0.0 | 0.0 | 0.0 | 0.0 | 0.0 | 0.0 | 0.0 | 0.0 | 0.0 |
| **IL6** | 0.0 | **99893.9** | -88.7 | **590.9** | **312.2** | -62.3 | **301.8** | **6425.2** | **1300.5** | -54.4 | **21.2** | -80.8 | **3064.9** | -17.4 | **842.7** | -76.8 | **391.0** | **47.9** | -47.4 |
| **IL7** | 0.0 | **1.8** | 0.0 | 0.0 | 0.0 | 0.0 | 0.0 | 0.0 | 0.0 | 0.0 | 0.0 | 0.0 | 0.0 | 0.0 | 0.0 | 0.0 | 0.0 | 0.0 | 0.0 |
| **IL9** | 0.0 | **184.4** | 0.0 | **108.0** | **21.9** | 0.0 | 0.0 | **63.9** | **30.5** | 0.0 | 0.0 | **48.9** | **120.8** | 0.0 | 0.0 | **23.1** | 0.0 | **56.4** | **151.6** |
| **IP10** | 0.0 | **27691.6** | **424.3** | -43.6 | -765.0 | -739.8 | -706.9 | **2220.2** | **1083.0** | -732.2 | -29.8 | -224.2 | -208.8 | -530.3 | **198.6** | -866.1 | -863.5 | -393.9 | -738.9 |
| **KC** | 0.0 | **99.5** | **5.2** | **5.6** | -7.0 | -4.7 | -7.0 | **24.8** | **22.7** | -3.9 | -0.3 | **2.4** | **2.3** | -4.3 | **3.6** | -5.2 | -7.0 | **1.0** | -1.7 |
| **LIF** | 0.0 | **640.6** | -208.1 | -142.2 | **238.8** | **114.3** | **121.7** | **148.0** | **237.8** | **44.4** | -20.7 | -204.3 | **375.4** | **136.0** | -24.6 | -176.0 | **131.3** | **94.7** | -175.6 |
| **LIX** | 0.0 | **1780.5** | -156.3 | **97.7** | **273.6** | -38.1 | **184.3** | **881.4** | **842.0** | -12.3 | **136.3** | -162.3 | **840.2** | **145.3** | **674.9** | -162.3 | **330.5** | **68.8** | -131.6 |
| **MCSF** | 0.0 | **9.6** | **5.3** | 0.0 | 0.0 | 0.0 | 0.0 | **2.5** | **1.3** | 0.0 | 0.0 | 0.0 | **3.1** | 0.0 | 0.0 | 0.0 | 0.0 | 0.0 | 0.0 |
| **MCP1** | 0.0 | **961.0** | -302.8 | -162.6 | -220.3 | -227.5 | -211.1 | **386.0** | -213.0 | -184.2 | -42.8 | -302.8 | -76.9 | -222.0 | -48.2 | -302.8 | -219.1 | -212.2 | -302.8 |
| **MIG** | 0.0 | **15.3** | **30.9** | 0.0 | 0.0 | 0.0 | 0.0 | 0.0 | 0.0 | 0.0 | 0.0 | 0.0 | 0.0 | 0.0 | 0.0 | 0.0 | 0.0 | 0.0 | 0.0 |
| **MIP1a** | 0.0 | **99943.3** | -33.2 | **173.7** | -6.0 | **113.1** | **24.4** | **99943.3** | **2562.8** | **148.7** | **409.4** | -10.0 | **30005.7** | **120.7** | **895.7** | -18.0 | **12.9** | **63.4** | -12.1 |
| **MIP1b** | 0.0 | **99931.9** | -64.7 | **150.3** | -7.2 | **109.3** | **54.7** | **29084.7** | **1329.5** | **169.3** | **263.4** | -68.0 | **1077.7** | **206.5** | **825.7** | -68.0 | **14.5** | **128.8** | -65.0 |
| **MIP2** | 0.0 | **96322.8** | -3367.3 | **638.5** | **96322.8** | -491.0 | **96322.8** | **96322.8** | **96322.8** | -289.0 | **96322.8** | -3545.9 | **96322.8** | **96322.8** | **96322.8** | -2664.4 | **96322.8** | **96322.8** | -2497.7 |
| **RANTES** | 0.0 | **99999.9** | **0.8** | **5.3** | 0.0 | **4.2** | 0.0 | **26.7** | **16.4** | **3.5** | **2.6** | 0.0 | **10.1** | **6.3** | **2.8** | 0.0 | 0.0 | **18.4** | 0.0 |
| **TNFa** | 0.0 | **75371.7** | -12.3 | -4.3 | **12.9** | 0.0 | **21.3** | **25613.4** | **368.9** | **13.5** | **29.2** | -10.4 | **25702.6** | **4.4** | **169.5** | **6.3** | **25.1** | **0.3** | **16.3** |
| **VEGF** | 0.0 | **8.0** | -69.3 | -51.3 | **53.7** | **28.6** | **41.8** | -41.8 | -33.0 | -26.3 | **2.9** | -69.3 | -10.8 | -18.8 | -44.3 | -66.3 | **90.4** | **62.6** | -64.5 |
| Mouse JAWSII monocytes/dendritic cells were stimulated with the 15 hit MCAs or with MPL, C48/80, or M7 as positive controls or in media without stimuli as a negative control for 24-hours. Cell supernatants were collected and measured for secreted cytokine production using a cytokine/chemokine multiplex assay. Cytokine/chemokine values for cells in media were subtracted from cells stimulated with the 15 MCAs or controls. Red values indicate a positive pg/ml value after pg/ml values in media was subtracted. | | | | | | | | | | | | | | | | | | | |

| **Table S5. Top 20 enhanced genes in the NALT after exposure to lead MCAs, M7 and MPL ranked based on p-value** | | | | | | | | | | | | | | | | | | | | |
| --- | --- | --- | --- | --- | --- | --- | --- | --- | --- | --- | --- | --- | --- | --- | --- | --- | --- | --- | --- | --- |
| **MPL** | | | **M7** | | | **R127655** | | | **R529877** | | | **ST027688** | | | **ST048871** | | | **ST101036** | | |
| **SYMBL** | **lo2FoldChange** | **adjusted-p** | **SYMBL** | **lo2FoldChange** | **adjusted-p** | **SYMBL** | **lo2FoldChange** | **adjusted-p** | **SYMBL** | **lo2FoldChange** | **adjusted-p** | **SYMBL** | **lo2FoldChange** | **adjusted-p** | **SYMBL** | **lo2FoldChange** | **adjusted-p** | **SYMBL** | **lo2FoldChange** | **adjusted-p** |
| **Ifit3** | 3.42 | 4.26E-27 | **Timp1** | 2.45 | 7.01E-11 | **Uap1l1** | 1.12 | 1.64E-10 | **Upp1** | 1.84 | 8.07E-11 | **NA** | 0.37 | 2.98E-04 | **Mad2l2** | 0.51 | 6.26E-11 | **Cxcl2** | 8.16 | 2.99E-30 |
| **Oasl1** | 3.13 | 2.44E-26 | **Cxcl2** | 4.93 | 8.02E-11 | **Cln5** | 0.54 | 4.59E-09 | **C2cd4a** | 2.86 | 1.45E-08 | **Dctpp1** | 0.75 | 5.79E-04 | **Hspa8** | 0.80 | 1.44E-10 | **Retnlg** | 6.60 | 1.86E-24 |
| **NA** | 3.00 | 6.85E-25 | **Clca2** | 2.41 | 3.01E-09 | **Pnpla7** | 0.79 | 4.92E-08 | **Gale** | 0.81 | 1.93E-08 | **Gm7120** | 1.14 | 9.50E-04 | **Timp1** | 2.37 | 1.44E-10 | **Il1r2** | 4.32 | 1.89E-23 |
| **Cxcl10** | 5.26 | 1.07E-24 | **Serpinb2** | 1.48 | 6.60E-09 | **Dpp7** | 0.58 | 1.03E-07 | **Psmd4** | 0.61 | 2.20E-08 | **Myh4** | 6.99 | 1.91E-03 | **Cxcl2** | 4.64 | 1.39E-09 | **Clec4d** | 5.13 | 7.61E-22 |
| **Ifi44** | 2.84 | 1.07E-24 | **Cxcl3** | 4.57 | 8.30E-09 | **Kng1** | 5.15 | 4.79E-07 | **A430005L14Rik** | 0.68 | 2.28E-08 | **Gale** | 0.52 | 1.91E-03 | **Tpd52** | 0.48 | 9.91E-09 | **Irg1** | 6.03 | 1.31E-21 |
| **Mx1** | 3.74 | 1.07E-24 | **Osm** | 3.37 | 8.38E-09 | **Gns** | 0.40 | 7.62E-07 | **Tapbpl** | 0.53 | 4.77E-08 | **Cck** | 1.12 | 2.40E-03 | **Urah** | 1.26 | 2.85E-08 | **Cxcr2** | 3.58 | 2.34E-21 |
| **I830012O16Rik** | 3.40 | 3.47E-24 | **Clec4d** | 3.15 | 6.25E-08 | **Tpp1** | 0.42 | 7.79E-07 | **Spdef** | 1.12 | 5.32E-08 | **0610007P14Rik** | 0.40 | 2.49E-03 | **Flot1** | 0.62 | 7.21E-08 | **Ccl2** | 4.19 | 8.71E-21 |
| **Rsad2** | 3.59 | 1.38E-23 | **Dctd** | 1.08 | 1.24E-07 | **Grina** | 0.57 | 9.65E-07 | **Nop16** | 0.67 | 7.60E-08 | **Wfdc2** | 0.80 | 2.95E-03 | **Epcam** | 0.85 | 9.44E-08 | **Il1b** | 4.69 | 8.71E-21 |
| **Ifit1** | 2.50 | 2.38E-23 | **Ppbp** | 2.18 | 1.30E-07 | **Trmt61a** | 0.77 | 1.49E-06 | **Dxo** | 0.58 | 1.27E-07 | **Ppia** | 0.46 | 3.20E-03 | **NA** | 0.84 | 1.62E-07 | **Rnd1** | 3.74 | 4.82E-20 |
| **Gbp3** | 2.37 | 2.91E-23 | **Tmem154** | 0.53 | 1.54E-07 | **Cyp51** | 0.65 | 1.85E-06 | **Gpx2** | 1.32 | 1.32E-07 | **Agr2** | 0.61 | 3.20E-03 | **Gm15459** | 0.91 | 2.03E-07 | **Cxcl1** | 6.13 | 8.32E-20 |
| **Isg15** | 3.01 | 5.73E-23 | **Irg1** | 3.61 | 1.54E-07 | **Plin3** | 0.55 | 4.52E-06 | **Manf** | 0.81 | 1.58E-07 | **Fbxl6** | 0.36 | 4.48E-03 | **Slc6a14** | 1.88 | 2.23E-07 | **Trem1** | 5.03 | 1.80E-19 |
| **Mx2** | 3.01 | 5.51E-22 | **Chil3** | 2.56 | 4.59E-07 | **Lrpprc** | 0.32 | 5.13E-06 | **Abhd11** | 0.71 | 1.97E-07 | **Pvalb** | 3.23 | 4.79E-03 | **Hspd1** | 0.45 | 2.30E-07 | **Mt1** | 2.94 | 5.00E-18 |
| **Slfn4** | 3.83 | 8.19E-22 | **Ccl3** | 2.69 | 6.73E-07 | **Naga** | 0.38 | 5.20E-06 | **Urah** | 1.23 | 3.39E-07 | **Aprt** | 0.56 | 5.08E-03 | **Clpp** | 0.63 | 2.57E-07 | **Il1rn** | 2.51 | 7.41E-18 |
| **Dhx58** | 2.04 | 2.95E-21 | **Ccl24** | 3.44 | 2.74E-06 | **Galc** | 0.49 | 5.69E-06 | **Cxcl2** | 4.15 | 4.07E-07 | **Gm7609** | 1.17 | 5.34E-03 | **Prr13** | 0.75 | 3.98E-07 | **Srgn** | 2.40 | 7.41E-18 |
| **Irf7** | 2.58 | 5.66E-21 | **Cd300ld** | 1.73 | 4.22E-06 | **Dram1** | 0.71 | 5.79E-06 | **Cda** | 1.19 | 4.07E-07 | **Commd3** | 0.34 | 5.88E-03 | **Osm** | 3.03 | 4.33E-07 | **Osm** | 4.66 | 1.47E-17 |
| **Ifih1** | 1.55 | 2.97E-18 | **Fcgbp** | 2.11 | 4.62E-06 | **Renbp** | 0.94 | 6.39E-06 | **Mcfd2** | 0.64 | 4.07E-07 | **Tspan8** | 0.65 | 6.12E-03 | **Hmga1** | 1.01 | 5.22E-07 | **Dusp5** | 2.38 | 1.47E-17 |
| **Gbp2** | 2.89 | 8.24E-18 | **Il1rl1** | 3.97 | 5.73E-06 | **Ppbp** | 1.85 | 6.61E-06 | **Ccl9** | 1.71 | 4.07E-07 | **Manf** | 0.50 | 6.76E-03 | **Wtip** | 0.67 | 5.31E-07 | **Adm** | 2.62 | 1.47E-17 |
| **Ifit2** | 1.74 | 3.80E-17 | **Cfi** | 1.78 | 6.22E-06 | **Cdk4** | 0.35 | 7.35E-06 | **Timp1** | 2.03 | 4.39E-07 | **Gmds** | 0.56 | 7.27E-03 | **A430005L14Rik** | 0.59 | 6.51E-07 | **Ccl3** | 4.16 | 1.47E-17 |
| **Parp9** | 1.27 | 4.36E-17 | **Ccl7** | 2.04 | 6.22E-06 | **P2rx4** | 0.29 | 9.75E-06 | **NA** | 0.70 | 4.39E-07 | **Tmed9** | 0.31 | 7.47E-03 | **Psmd4** | 0.52 | 6.51E-07 | **Ppp1r15a** | 1.91 | 1.68E-17 |
| **Ccl2** | 3.61 | 6.50E-17 | **Ccl4** | 2.87 | 6.22E-06 | **Hist1h1d** | 1.33 | 1.17E-05 | **Flot1** | 0.61 | 4.47E-07 | **Ica1** | 0.37 | 8.07E-03 | **Serpinb1a** | 1.42 | 7.86E-07 | **Upp1** | 2.15 | 3.74E-17 |

| **Table S6. Top 20 inhibited genes in the NALT after exposure to 5 Lead MCAs, M7 and MPL ranked based on p-value** | | | | | | | | | | | | | | | | | | | | |
| --- | --- | --- | --- | --- | --- | --- | --- | --- | --- | --- | --- | --- | --- | --- | --- | --- | --- | --- | --- | --- |
| **MPL** | | | **M7** | | | **R127655** | | | **R529877** | | | **ST027688** | | | **ST048871** | | | **ST101036** | | |
| **SYMBL** | **lo2FoldChange** | **adjusted-p** | **SYMBL** | **lo2FoldChange** | **adjusted-p** | **SYMBL** | **lo2FoldChange** | **adjusted-p** | **SYMBL** | **lo2FoldChange** | **adjusted-p** | **SYMBL** | **lo2FoldChange** | **adjusted-p** | **SYMBL** | **lo2FoldChange** | **adjusted-p** | **SYMBL** | **lo2FoldChange** | **adjusted-p** |
| **NA** | -4.15 | 2.42E-15 | **NA** | -3.62 | 8.02E-11 | **Fggy** | -0.78 | 8.32E-13 | **Col14a1** | -1.03 | 8.89E-11 | **Soga1** | -0.62 | 6.37E-06 | **Col14a1** | -0.96 | 1.44E-10 | **Gm15883** | -2.87 | 1.80E-19 |
| **Bpifb9b** | -12.50 | 3.82E-09 | **Ksr2** | -1.54 | 1.80E-07 | **Olfr544** | -2.54 | 1.13E-09 | **Stab2** | -1.43 | 2.35E-10 | **Ptpn13** | -0.39 | 9.98E-06 | **Ccdc176** | -1.05 | 5.32E-10 | **6330403L08Rik** | -0.96 | 3.38E-14 |
| **Nrp1** | -0.59 | 4.86E-07 | **Nrp1** | -0.56 | 1.40E-05 | **Cdh4** | -1.02 | 5.59E-09 | **Fmo2** | -1.26 | 6.45E-09 | **Sh3pxd2a** | -0.34 | 1.46E-05 | **Olfr1206** | -22.74 | 2.85E-08 | **Gm15675** | -2.08 | 2.01E-12 |
| **Ksr2** | -1.28 | 9.68E-06 | **Bpifb9a** | -5.89 | 2.37E-05 | **Ndp** | -0.94 | 3.70E-08 | **Npnt** | -0.90 | 6.56E-09 | **Gm5884** | -1.88 | 1.46E-05 | **Fmo2** | -1.12 | 6.92E-08 | **Cfap46** | -1.52 | 2.46E-12 |
| **Bpifb5** | -5.74 | 1.10E-05 | **Soga1** | -0.53 | 3.37E-05 | **Slc17a6** | -1.10 | 3.70E-08 | **Epha5** | -1.11 | 6.56E-09 | **Epha5** | -0.88 | 1.46E-05 | **Syne3** | -0.88 | 3.30E-07 | **Grhl2** | -0.97 | 2.63E-12 |
| **Rpl38-ps2** | -2.05 | 1.30E-05 | **Fbn2** | -0.82 | 3.60E-05 | **Rgs7** | -1.65 | 3.70E-08 | **Ccdc176** | -1.00 | 4.26E-08 | **Gm7964** | -0.43 | 9.70E-05 | **Npnt** | -0.76 | 3.30E-07 | **Proser3** | -1.26 | 2.85E-12 |
| **Gm10167** | -1.70 | 4.05E-05 | **Etl4** | -0.37 | 3.96E-05 | **Dpys** | -1.47 | 3.70E-08 | **Sh3pxd2a** | -0.41 | 4.26E-08 | **Gm9762** | -1.48 | 1.85E-04 | **Itpr1** | -0.58 | 4.05E-07 | **Muc5ac** | -4.15 | 1.23E-11 |
| **Bpifb9a** | -5.43 | 4.99E-05 | **Hcn1** | -0.94 | 4.29E-05 | **Etv4** | -1.28 | 3.70E-08 | **Soga1** | -0.69 | 7.60E-08 | **Etl4** | -0.37 | 1.85E-04 | **Kitl** | -0.66 | 4.33E-07 | **Ccdc176** | -1.10 | 5.51E-11 |
| **Gm5884** | -1.58 | 6.39E-05 | **Gm5884** | -1.67 | 5.46E-05 | **Gm6878** | -1.42 | 4.92E-08 | **Nrp1** | -0.67 | 2.44E-07 | **Akap13** | -0.37 | 2.65E-04 | **Aldh1a1** | -1.34 | 6.51E-07 | **Lrrc71** | -2.34 | 7.18E-11 |
| **Olfr911-ps1** | -2.98 | 6.70E-05 | **Bpifb5** | -5.56 | 7.47E-05 | **F8** | -0.96 | 5.86E-08 | **Tenm3** | -0.65 | 3.39E-07 | **Bpifb5** | -5.63 | 2.65E-04 | **Antxr1** | -0.55 | 6.68E-07 | **Fam161a** | -1.35 | 1.10E-10 |
| **Cdh4** | -0.70 | 7.96E-05 | **Zfp608** | -0.39 | 1.09E-04 | **Kirrel3** | -0.94 | 1.29E-07 | **Ksr2** | -1.57 | 4.07E-07 | **Ksr2** | -1.24 | 2.98E-04 | **Akap13** | -0.43 | 1.06E-06 | **Ccdc17** | -1.91 | 1.12E-10 |
| **Tex15** | -0.77 | 9.04E-05 | **Cep83** | -0.46 | 1.48E-04 | **Acsl6** | -1.02 | 1.29E-07 | **Ppap2b** | -0.74 | 4.39E-07 | **D630045J12Rik** | -0.64 | 5.79E-04 | **Soga1** | -0.60 | 1.06E-06 | **E030010N08Rik** | -2.18 | 1.14E-10 |
| **Hcn1** | -0.86 | 1.02E-04 | **Bpifb9b** | -9.29 | 1.55E-04 | **Mmel1** | -1.36 | 1.34E-07 | **Kat6b** | -0.50 | 5.64E-07 | **Lcor** | -0.76 | 5.79E-04 | **Adamtsl1** | -0.82 | 1.31E-06 | **Wdr95** | -2.26 | 1.95E-10 |
| **Fbn2** | -0.73 | 1.15E-04 | **Aldh1a1** | -1.09 | 1.93E-04 | **Sec14l3** | -1.51 | 1.34E-07 | **Syne3** | -0.89 | 1.12E-06 | **Klhl11** | -0.94 | 9.48E-04 | **Ksr2** | -1.42 | 1.49E-06 | **Ttc16** | -1.65 | 3.40E-10 |
| **Olfr286** | -5.42 | 1.54E-04 | **NA** | -2.82 | 1.97E-04 | **Cyp3a13** | -1.28 | 1.60E-07 | **Enah** | -0.32 | 1.14E-06 | **Wasf2** | -0.28 | 9.50E-04 | **Cdon** | -0.62 | 1.49E-06 | **NA** | -1.46 | 4.78E-10 |
| **Gm9762** | -1.28 | 2.08E-04 | **Gm18588** | -1.73 | 2.58E-04 | **Gm13185** | -1.88 | 1.70E-07 | **Slc7a2** | -0.61 | 1.20E-06 | **Rpl38-ps2** | -1.88 | 9.88E-04 | **Epha5** | -0.87 | 1.87E-06 | **Myo5c** | -0.74 | 5.68E-10 |
| **Gatsl2** | -0.47 | 2.36E-04 | **Gm10123** | -1.02 | 2.58E-04 | **Rtp1** | -0.94 | 1.70E-07 | **Arhgap31** | -0.45 | 1.62E-06 | **Rpl17-ps10** | -1.87 | 1.06E-03 | **Arhgap32** | -0.43 | 1.98E-06 | **Srgap2** | -0.57 | 6.27E-10 |
| **Olfr781** | -2.73 | 2.40E-04 | **Rpl38-ps2** | -1.87 | 2.94E-04 | **Brdt** | -0.70 | 2.41E-07 | **Abi3bp** | -0.89 | 1.73E-06 | **Hcfc1** | -0.33 | 1.06E-03 | **Wdr6** | -0.46 | 1.99E-06 | **4833428L15Rik** | -2.76 | 6.42E-10 |
| **Ppargc1a** | -0.67 | 2.69E-04 | **Mgea5** | -0.34 | 3.31E-04 | **Il33** | -1.08 | 2.41E-07 | **Plekhg1** | -0.57 | 2.40E-06 | **Bpifb9a** | -5.08 | 1.68E-03 | **Ptprm** | -0.64 | 2.32E-06 | **Disp1** | -0.77 | 7.53E-10 |
| **Zfp608** | -0.36 | 2.74E-04 | **Kirrel2** | -1.32 | 3.33E-04 | **Lingo2** | -1.32 | 3.21E-07 | **Syne1** | -0.95 | 2.71E-06 | **Gm10123** | -0.99 | 1.68E-03 | **Ptprn2** | -1.41 | 2.73E-06 | **Cbx6** | -0.61 | 8.50E-10 |
